# Distributing tasks via multiple input pathways increases cellular survival in stress

**DOI:** 10.7554/eLife.21415

**Published:** 2017-05-17

**Authors:** Alejandro A Granados, Matthew M Crane, Luis F Montano-Gutierrez, Reiko J Tanaka, Margaritis Voliotis, Peter S Swain

**Affiliations:** 1 SynthSys - Synthetic and Systems Biology, University of Edinburgh, Edinburgh, United Kingdom; 2 Department of Bioengineering, Imperial College London, London, United Kingdom; 3 School of Biological Sciences, University of Edinburgh, Edinburgh, United Kingdom; 4 Department of Mathematics and Living Systems Institute, College of Engineering, Mathematics, and Physical Sciences, University of Exeter, Exeter, United Kingdom; Weizmann Institute of Science, Israel

**Keywords:** cellular perception, speed-accuracy trade-off, MAP kinase, signal transduction, stress response, microfluidics, *S. cerevisiae*

## Abstract

Improving in one aspect of a task can undermine performance in another, but how such opposing demands play out in single cells and impact on fitness is mostly unknown. Here we study budding yeast in dynamic environments of hyperosmotic stress and show how the corresponding signalling network increases cellular survival both by assigning the requirements of high response speed and high response accuracy to two separate input pathways and by having these pathways interact to converge on Hog1, a p38 MAP kinase. Cells with only the less accurate, reflex-like pathway are fitter in sudden stress, whereas cells with only the slow, more accurate pathway are fitter in increasing but fluctuating stress. Our results demonstrate that cellular signalling is vulnerable to trade-offs in performance, but that these trade-offs can be mitigated by assigning the opposing tasks to different signalling subnetworks. Such division of labour could function broadly within cellular signal transduction.

**DOI:** http://dx.doi.org/10.7554/eLife.21415.001

## eLife digest

The faster we do tasks the harder it is to do them well. For example, when we wish to judge if, say, a cup, is too hot, we first quickly withdraw our hand after touching it: we know that the cup is hot but not how much. Next we hold a finger against the cup to accurately judge its temperature. Such speed-accuracy trade-offs are studied widely in fields ranging from neuroscience to engineering, but their consequences for single cells are unknown. This is despite the fact that when cells are exposed to stress they must respond both quickly (to survive) and accurately (to reduce how many resources they consume).

One way of stressing yeast cells is to place them in a syrupy substance called sorbitol. This causes the cells to lose water, shrink in size, and launch a stress response to regain volume. If the cells respond inappropriately to the situation, they may die.

The signalling network that produces the stress response is unusual in that it has a Y-shaped structure, where the two ‘arms’ of the Y are the input pathways. Although it was known that one input pathway responds to stress faster than the other, the advantages of having two inputs in the signalling network were not understood. Granados, Crane et al. thought that the differences in speed and the Y-shaped structure could allow the cell to respond to stress with both speed and accuracy.

To investigate this theory, Granados, Crane et al. used a microscope to study individual yeast cells that had been exposed to sorbitol. Combining these results with a mathematical model of the cell signalling network revealed that a mutant yeast cell that only has one of the input pathways specializes in speed but is inaccurate, similar to a reflex-like response. In contrast, a mutant with only the other pathway specializes in accuracy, being slower but matching the level of the cell’s response to the level of stress placed on it. This trade-off is reflected in rates of cell survival: the first mutant survives best in sudden shocks of stress; the second mutant survives best in gradually increasing stress. Normal yeast cells that have both input pathways survive more often than either mutant.

Overall, the results presented by Granados, Crane et al. reveal principles behind cellular decision-making that should hold true in more complex organisms and could be exploited by synthetic biologists to programme cells with new behaviours.

**DOI:** http://dx.doi.org/10.7554/eLife.21415.002

## Introduction

Cells must adapt to changes in their environment and to do so specialise their response to the nature of the signal being detected. Improving performance in one task, however, often undermines performance in another (Pareto, 1896). In engineering, for example, it is well-known that fast responses have lower accuracy and that higher amplifications can cause overshooting and unintended oscillations (Astrom and Murray, 2008).

At the cellular level, we expect signal transduction has evolved to reduce such trade-offs because performance in, for example, both speed and accuracy are likely to be under selection (Shoval et al., 2012; Lan et al., 2012; Siggia and Vergassola, 2013). In particular, stress responses can not only be a life-and-death situation where a too slow response is fatal, but also often lead to the consumption of substantial cellular resources so that cells must accurately coordinate their response with the stress (López-Maury et al., 2008; Perkins and Swain, 2009). To maintain accuracy, we can think of cells having to continuously match the degree of activation of signalling networks with both their internal state and with the magnitude and type of extracellular signals. By doing so, cells can then correctly ‘interpret’ the environment and launch and modify the appropriate response at the appropriate level.

To understand how cells mitigate trade-offs in signalling, we turned to one of the most studied eukaryotic stress responses: hyperosmotic stress in budding yeast. Following an abrupt increase in environmental osmolarity, yeast cells can shrink in seconds (Hohmann, 2002) and must therefore respond quickly. Their response though is metabolically costly, involving the synthesis of the osmoprotectant glycerol, and inaccurate hyperactivation of the signalling network can be highly deleterious (Tao et al., 1999; Mitchell et al., 2015).

**Figure 1.**
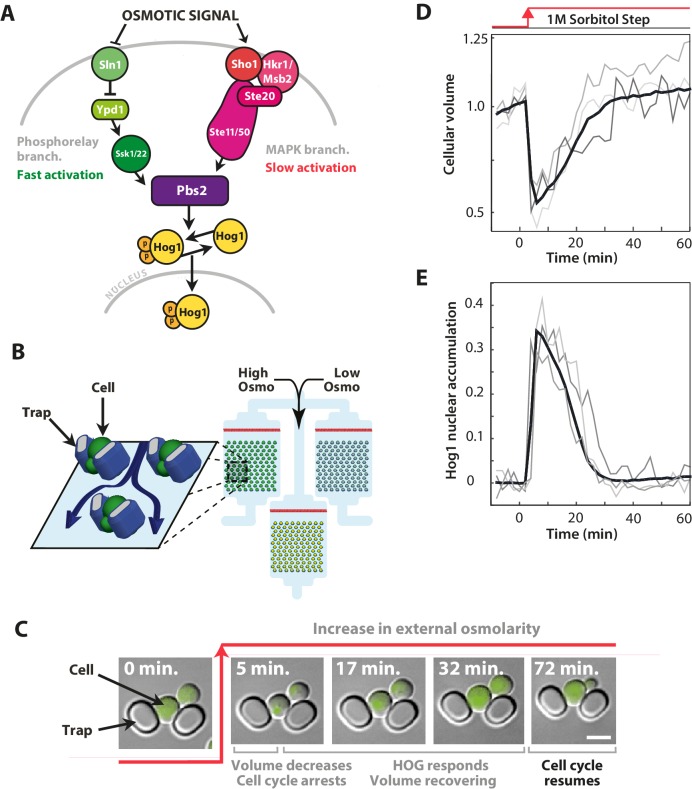
The signalling network in budding yeast that responds to hyperosmotic stress has two input pathways, both activating Pbs2 and Hog1, and its response can be quantified in single cells using the nuclear localisation of Hog1. (**A**) Two input branches regulate the activity of the Hog1 kinase. The Sln1 (green) branch is a bacterial-like phosphorelay. The Sho1 (red) branch is a MAP kinase cascade, which is tethered to the membrane by the sensors Sho1 and Msb2. (**B**) A schematic showing the operation of the ALCATRAS microfluidic device (Crane et al., 2014). Single cells are confined between PDMS traps (blue) and exposed to changes in osmolarity. To ensure all strains experience identical environments, they are loaded into separate chambers of the same device. (**C**) When exposed to hyperosmotic stress, cellular volume shrinks, Hog1 undergoes nuclear translocation, and cells arrest. Growth typically resumes once the volume has recovered. (**D,E**) A reduction in volume causes Hog1 to translocate within minutes and the recovery of the volume correlates with levels of nuclear Hog1. The average of the cell population (n=356) and three single-cell traces selected at random are shown. **DOI:** http://dx.doi.org/10.7554/eLife.21415.003

When the osmolarity of the environment increases, yeast activate a p38 kinase, Hog1, to launch the stress response. The Hyper-Osmolarity-Glycerol (HOG) network has a Y-shaped structure with two input pathways both converging on Pbs2, a MAP kinase kinase (Figure 1A). One branch of the Y, the Sln1 pathway, uses a two-component phosphorelay, analogous to those in bacteria, to propagate the signal (Posas et al., 1996; Posas and Saito, 1998); the other, the Sho1 branch, uses protein kinases, similar to signalling in higher organisms (Posas and Saito, 1997; Tatebayashi et al., 2006). Once activated, Pbs2 in turn activates Hog1 via phosphorylation (Brewster et al., 1993). Similarly to MAP kinases in mammalian cells, Hog1 can then translocate into the nucleus. Upon activation, Hog1 causes an increase in the intracellular concentration of glycerol, yeast’s main osmoprotectant, in two ways (Saito and Posas, 2012): first, through cytosolic changes, such as diverting glycolysis towards synthesizing glycerol and closing channels that export glycerol, and, second, through altering gene expression to increase the numbers of enzymes involved in glycerol synthesis. As levels of intracellular glycerol increase, water returns to the cell, and the cellular volume expands. This increase in volume reduces signalling through the HOG network and the levels of activation of Hog1.

Despite these discoveries, the advantage of having two input pathways in the HOG network is still not understood (Tanaka et al., 2014; Brewster and Gustin, 2014). The two branches of the Y may sense stress differently (Reiser et al., 2003; Tanaka et al., 2014) and are known to operate at different time-scales (Maeda et al., 1995; Hersen et al., 2008): the Sln1 branch being faster than the Sho1 branch. These different response times imply that each pathway has the potential to respond distinctly to input signals (Behar et al., 2007) and hence generate distinct dynamics of volume recovery. Mutants that have only one branch of the Y have been created (Maeda et al., 1995; Reiser et al., 2003; Hersen et al., 2008; Macia et al., 2009; Schaber et al., 2012; English et al., 2015), but there is no reported phenotype for strains having only the Sln1 branch, and the Sho1 pathway is often considered redundant (Klipp et al., 2005; Muzzey et al., 2009).

We hypothesized that the Y-shaped structure could allow the cell to respond to stress with both speed and accuracy. Our approach was to characterize the behaviour of both Hog1 and cellular volume at the single-cell level in the wild-type and in mutants with only one of the input pathways. With both types of single-cell measurements, we can quantify accuracy by the statistical dependency between the dynamics of cellular volume and the dynamics of nuclear Hog1. We show that each input pathway specializes to a particular task and that by having the two pathways the wild-type is both fast and accurate over a wide range of dynamic environments.

## Results

To understand how the HOG network might mitigate trade-offs in performance, we measured the extent of cellular stress by the reduction of the cellular volume and the extent of activation of the HOG network by the degree of nuclear localization of Hog1. The nuclear level of Hog1 has long been used as a read-out of the HOG network’s response (Mettetal et al., 2008; Muzzey et al., 2009; Pelet et al., 2011; Babazadeh et al., 2013; Mitchell et al., 2015) and is strongly correlated with Hog1 phosphorylation (Reiser et al., 1999).

We measured the dynamics of Hog1 in the wild-type strain and in two established mutants (Hersen et al., 2008; English et al., 2015)—a ‘fast’ mutant with only the Sln1 branch (deletion of Ste11) and a ‘slow’ mutant with only the Sho1 branch (deletion of Ssk1)—at the same time and in stress with identical dynamics (Figure 1B) and quantified single-cell responses (Figure 1C–E). To impose osmotic stress, we use sorbitol (Hersen et al., 2008), which unlike salts does not apply any additional stress from toxic cations (Posas et al., 2000).

### Mutants with just one of the input pathways have different accuracy

**Figure 2.**
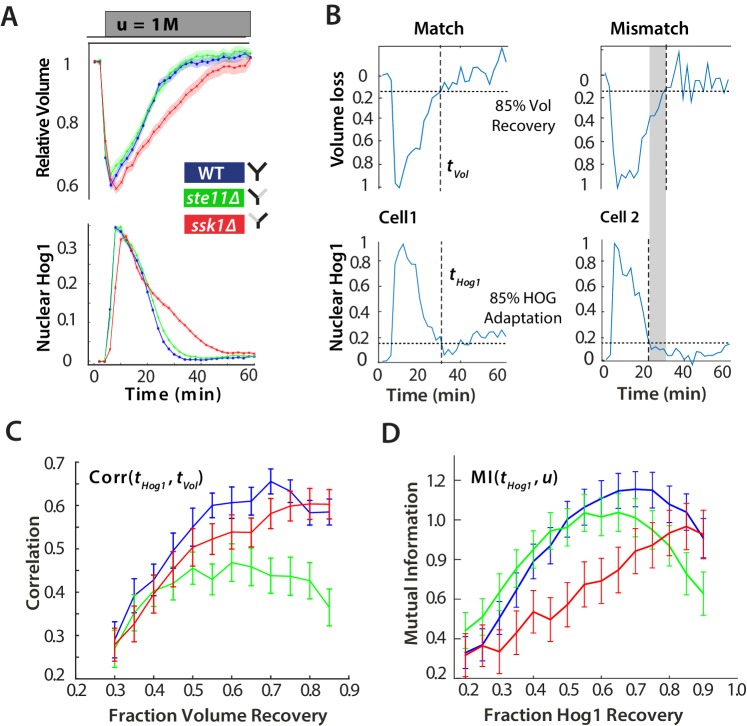
For hyperosmotic stress, accuracy can be quantified as the statistical dependency between the dynamics of Hog1 and the dynamics of volume recovery. (**A**) Characterization of the wild-type (WT) and mutant strains in response to a 1 M sorbitol step. Colours here and in all following figures: blue (WT); green (fast mutant—ssk1Δ); red (slow mutant—ste11Δ). Mean responses are shown and error bars are SEM. See also Video 1. (**B**) Normalized response from wild-type cells to illustrate the degree of matching between the time of adaptation of Hog1 (the time for nuclear Hog1 to undergo a 85% decrease from its maximum) and the time of volume recovery (the time for the volume to undergo a 85% increase from its minimum). (**C**) Accuracy is the correlation between the adaptation times and is lowest for the fast mutant in late stages of the volume recovery (data from six experiments with at least 500 cells per strain; Figure 2—figure supplement 1). Error bars are 95% confidence intervals for the mean calculated by bootstrapping. (**D**) Adaptation of Hog1 in single cells becomes less sensitive to the magnitude of the stress in the fast mutant. The mutual information between the distributions of adaptation times of Hog1 and the magnitude of the steps from four experiments shows that the fast mutant becomes the least informative late in adaptation explaining the drop in correlation in **C**. Error bars are 95% credible intervals for the mean calculated by bootstrapping. Differences between strains are therefore at a 5% significance level when the error bars do not overlap. **DOI:** http://dx.doi.org/10.7554/eLife.21415.004

In steps of hyperosmotic stress, by far the most common type of input so far investigated (Saito and Posas, 2012), the fast (Sln1 only) mutant has been reported to perform almost identically to wild-type. We first verified that the two mutants, each with one of the branches of the Y, behave as expected (Maeda et al., 1995; Hersen et al., 2008; Macia et al., 2009). Indeed, in steps, the mean response of Hog1 in the fast mutants is equivalent to wild-type cells, but the slow mutant has typically longer response times and a lower maximum level of nuclear localization (Figure 2A).

**Figure 2—figure supplement 1.**
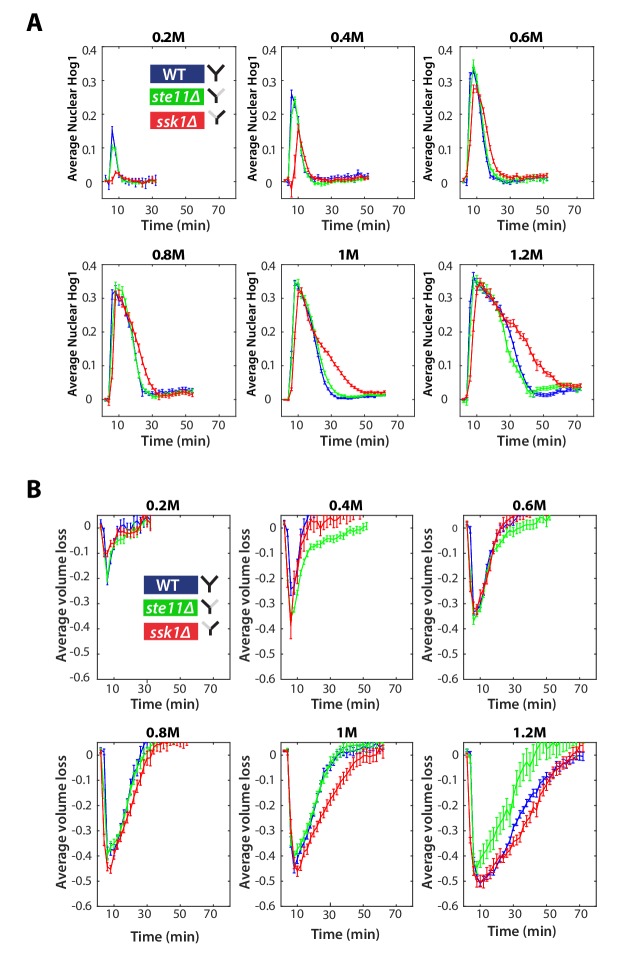
The Hog1 and volume response for wild-type and mutants in steps. (**A**) Step data used in Figure 2C with the sorbitol concentrations given above each panel. Average and SEM error bars are shown. Numbers of cells are listed in order of wild-type, ste11∆, ssk1∆ for each experiment (n = 78, 112, 94 for 0.2 M; n = 116, 140, 87 for 0.4 M; n = 105, 123, 113 for 0.6 M ; n = 82, 81, 87 for 0.8 M; n = 192, 148, 125 for 1.0 M; n = 133, 89, 94 for 1.2 M). (**B**) Average volume traces for the experiments shown in A. Error bars are SEM. **DOI:** http://dx.doi.org/10.7554/eLife.21415.005

**Figure 2—figure supplement 2.**
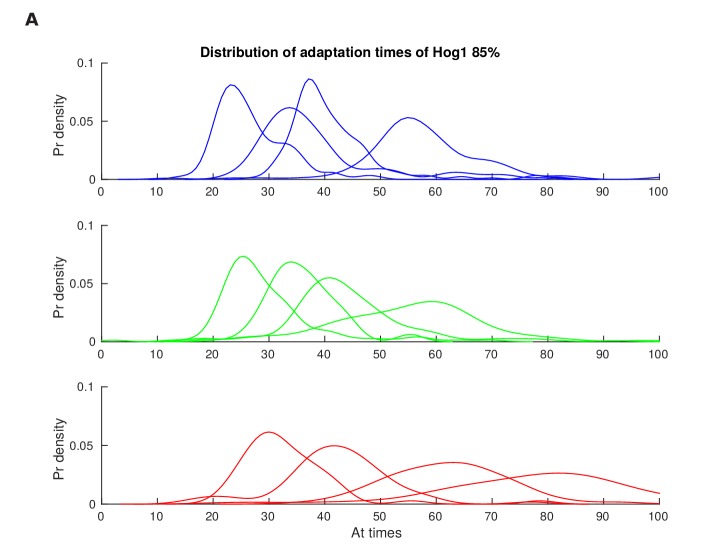
Distributions of the adaptation time of Hog1 for different step inputs. Single-cell distributions of the adaptation time of Hog1 (time to adapt to 85% of the maximum value) for step inputs of 0.6, 0.8, 1 and 1.2 M sorbitol. Adaptation times were found from the experiments of Figure 2—figure supplement 1 and the distributions used to calculate the mutual information in Figure 2D. **DOI:** http://dx.doi.org/10.7554/eLife.21415.006

**Video 1.** Nuclear localization of Hog1-GFP in a step of 1M sorbitol for the wild-type and two mutants (related to Figure 2). Overlay of DIC and fluorescence microscopy images showing cells trapped between two pillars in the ALCATRAS microfluidics device. **DOI:** http://dx.doi.org/10.7554/eLife.21415.007

Considering the reduction in cellular volume, the wild-type and fast response are again almost identical on average, and the longer response time of the slow mutant is reflected in a slower volume recovery (Figure 2A), particularly in larger steps (Figure 2—figure supplement 1). In all strains, the mean volume and the mean level of nuclear Hog1 simultaneously go to zero (Figure 2A).

At the single-cell level, however, this picture changes (Figure 2B). We quantified the degree to which the cellular response matches the cellular volume—the accuracy of the response—by the statistical dependency (the Pearson correlation) between the time of adaptation of Hog1 and the time of adaptation of the volume. Cells differ in their internal states, for example in their intracellular levels of glycerol, and so the recovery of the volume reports the extent of the subjective stress experienced by each cell. To normalize between cells and for the magnitude of the step, the correlation is calculated for different fractions of the recovery of the volume (Figure 2C). Although the accuracy of all strains increases as the volume recovers, it is the wild-type and the slow mutant that behave similarly, and the correlation for the fast mutant remains consistently lower than the wild-type.

This discrepancy between the mean (Figure 2A) and the single-cell results (Figure 2C) implies that the Hog1 behaviour in the fast mutant during adaptation is more noisy than the wild-type. The variation can be quantified by the statistical dependency (the mutual information) between the adaptation times of Hog1 in single cells and the magnitude of the stress (Figure 2D). A higher mutual information implies that there is less overlap between the distributions of adaptation times for each stress (Figure 2—figure supplement 2) and therefore that the adaptation time of a typical Hog1 response is different for different levels of stress. As Hog1 adapts, Hog1 in the fast mutant becomes less informative on the level of stress and its distribution of adaptation times is broader than the wild-type for some stresses.

The two pathways therefore have contrasting behaviors: the slow pathway has a slower mean response of Hog1 but is almost as accurate as the wild-type at long times, and the fast pathway although responding the same as the wild-type on average is inaccurate at the single-cell level. Our results suggest that maintaining accuracy is principally addressed by the slow pathway, which best correlates the dynamics of Hog1 with the dynamics of the cellular volume in individual cells.

### Sensitivity to the system’s negative integral feedback affects accuracy

The adaptation of Hog1 and the adaptation of the volume are connected by negative feedback (Muzzey et al., 2009). This feedback acts through intracellular glycerol. Higher intracellular concentration of glycerol cause water to move into the cell and the resulting increase in volume reduces the level of activation of the HOG network. The rate of increase in glycerol is expected to depend on the time-integral of nuclear Hog1 (Muzzey et al., 2009; English et al., 2015), and the feedback is therefore called integral feedback (Astrom and Murray, 2008).

We reasoned that if the slow mutant is able to gradually increase its accuracy (Figure 2C) then the slow pathway should be more sensitive to the integral feedback. Indeed, the slow pathway unlike the fast pathway is known to have multiple types of osmo-sensors (Tanaka et al., 2014) and so may better sense the increase in volume resulting from the increase in glycerol.

**Figure 3.**
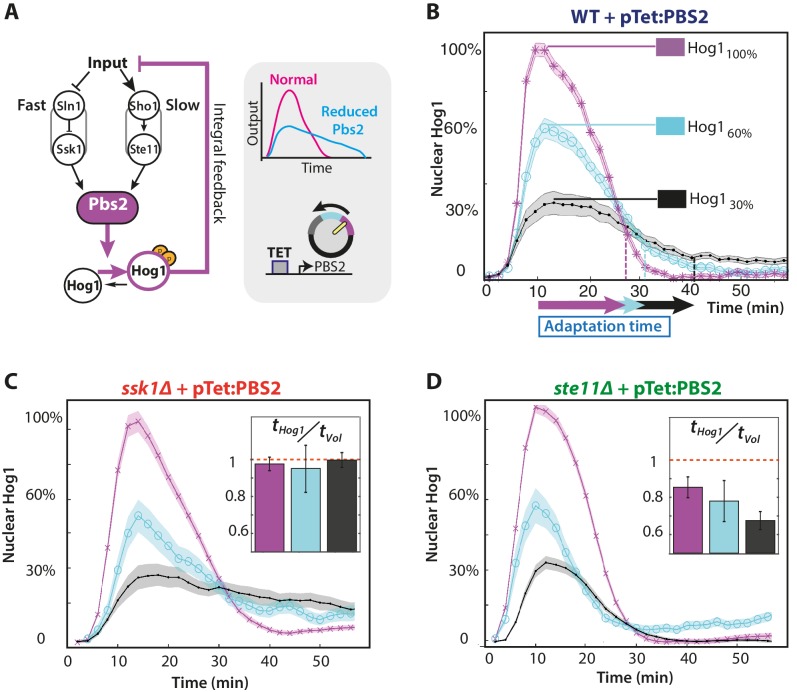
The slow pathway specializes in matching Hog1 dynamics to volume recovery by being most sensitive to the network’s integral feedback. (**A**) Before reaching Hog1, the signals from each pathway are transduced through Pbs2, and we perturb the HOG network by controlling PBS2 expression through a TET inducible promoter. Reduced induction of PBS2 compromises the network and decreases the maximum activity of Hog1. This reduction in activity should be compensated by the system’s integral feedback lengthening the nuclear residence of Hog1 (box inset). (**B**) Measuring relative to unperturbed Pbs2, under-expression of Pbs2 reduces the amplitude of mean Hog1 nuclear localization, but increases its adaptation time on average in wild-type cells (1 M step; arrows indicate time for 85% adaptation). (**C,D**) Mean Hog1 dynamics for the slow and fast mutants show that only the slow mutant extends the adaptation time of Hog1 like the WT. Insets: Mean ratio for three experiments of the adaptation time of Hog1 to the adaptation time of the volume in single cells. Error bars are SEM. **DOI:** http://dx.doi.org/10.7554/eLife.21415.008

To determine the sensitivity of each pathway to the feedback, we exogenously perturbed the level of activation of the network to measure the extent to which each pathway can compensate for the perturbation. If Hog1 activity in the nucleus is reduced and the network is sensitive to the integral feedback, the system will compensate by increasing the time spent by Hog1 in the nucleus (Figure 3A) (Mettetal et al., 2008; Zi et al., 2010). We therefore expect the wild-type and the slow mutant, but not the fast, to maintain accuracy in compromised networks.

**Figure 3—figure supplement 1.**
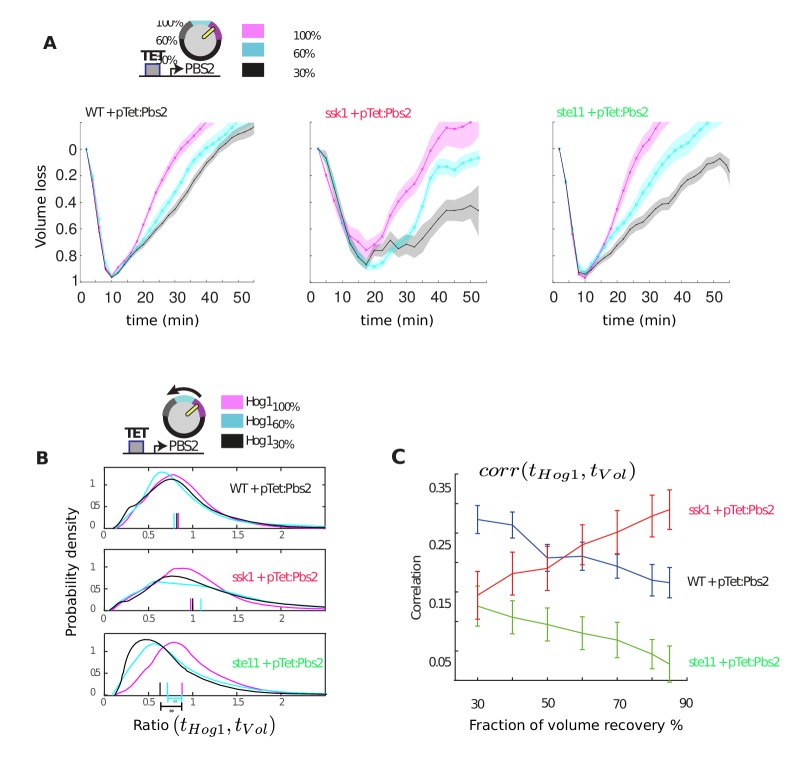
Only the wild-type and slow mutant can compensate when Pbs2 levels are reduced by increasing the adaptation time of Hog1. (**A**) Average volume as a function of time in the three strains for different levels of Pbs2 repression. The time to recover the volume is extended in all strains for reduced levels of Pbs2. (**B**) Distributions of the ratio between the adaptation time of Hog1 and the adaptation time of the volume in single cells for different levels of Pbs2. Each distribution comprises at least 200 cells from three independent experiments (27 experiments in total). The median is indicated by a coloured line on the x-axis. For the fast mutant, reduced levels of Pbs2 result in premature adaptation of Hog1 relative to the volume recovery (p<10^−6^: two-sided Wilcoxon rank sum test for equal medians and indicated by asterisks). For wild-type and the slow mutant, the ratio of the time of adaptation of Hog1 adaptation to the time of adaptation of the volume is not significantly affected by reduced Pbs2 levels (p>0.1). (**C**) The correlation between adaptation time of Hog1 and the time for volume recovery as a percentage of volume recovery calculated by pooling together the data from B (cf. Figure 2C). **DOI:** http://dx.doi.org/10.7554/eLife.21415.009

By exogenously decreasing levels of the MAP kinase kinase Pbs2, which lies downstream of both pathways (Figure 3A), we perturbed the network’s activity and showed that the slow pathway is most sensitive to the integral feedback. Decreasing expression of PBS2 reduces the maximum level of Hog1 localization but increases its adaptation time in both the wild-type and slow mutant (Figure 3B and C). In contrast, for the fast pathway, there is little increase in the adaptation time (Figure 3D). Similarly, there is a corresponding change in the speed of the response of the slow, but not the fast, pathway indicating that the slow pathway is better coupled to the dynamics of the integral feedback. Both the ratio of adaptation time of Hog1 to the adaptation time of the volume and the accuracy decreases significantly only for the fast mutant (Figure 3D inset with p<10^−6^ using a two-sided Wilcoxon rank sum test for equal medians calculated by pooling distributions from single cells).

To reduce metabolic costs, cells should respond accurately to stress, matching the dynamics of their response with the dynamics of the stress and of their internal states. Our results are consistent with this task being principally performed by the slow pathway because of its stronger coupling to the system’s integral feedback.

The insensitivity of the adaptation time of Hog1 and the sensitivity of the adaptation time of the volume for the fast mutant in these experiments (Figure 3D; Figure 3—figure supplement 1) suggest the existence of a mechanism within the fast pathway that decreases signalling of Hog1 before the volume completely recovers. This additional adaptation within the pathway to a step of stress implies that the fast pathway has the potential to respond to the time-derivative of its input (Block et al., 1983; Behar et al., 2007; Alon, 2007). A derivative response, referred to as derivative action, is often used in engineering to improve performance by predicting the future behaviour of the input (Astrom and Murray, 2008).

### Ramp inputs indicate derivative action within the fast pathway

**Figure 4.**
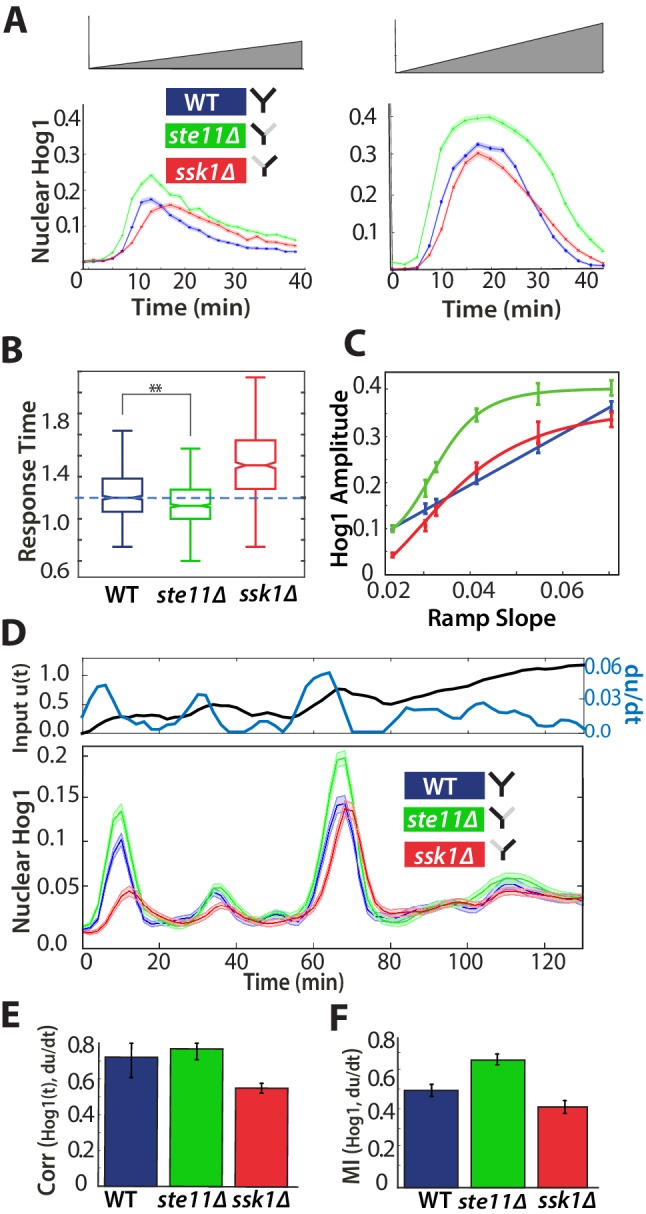
A component of the fast pathway that responds to the time-derivative of the input enables its high speed. (**A**) The Hog1 trajectory in the fast mutant overshoots the Hog1 trajectories of both the wild-type and the slow mutant in ramp inputs (two examples with different slopes of approximately 0.03 M min${}^{-1}$ and 0.06 M min${}^{-1}$). The mean response is shown and error bars are SEM. (**B**) Distributions of response times relative to the wild-type for six different ramps (Figure 4—figure supplement 1) shows that the fast mutant is even quicker than the wild-type on average (p-value $<10^{-6}$ using a t-test for distributions with at least 600 cells per strain). (**C**) The average amplitude of the Hog1 response for the fast mutant consistently overshoots the wild-type for ramp inputs, which responds linearly to the slope of the ramp. (**D**) An input with a fluctuating time-derivative shows the average Hog1 response of the fast mutant consistently over-shooting the wild-type. Errors are SEM. (**E**) The average of the single-cell cross-correlations of the trajectories of Hog1 with the trajectory of the (smoothed) time-derivative of the input shows that the high correlation of the wild-type comes from the fast and not the slow pathway (average of three independent experiments with fluctuating ramps and error bars as SD; p-value $<10^{-6}$ using a t-test on pooled single-cell data from the three experiments). (**F**) The mutual information between the time-derivative of the input in D and the level of Hog1 at each time point shows that the fast mutant best predicts the time-derivative (at 5% significance level calculated using credible intervals of the median). **DOI:** http://dx.doi.org/10.7554/eLife.21415.010

A system that only senses the time-derivative of an input should continually respond to an input that ramps linearly from low to high values because such an input has a constant time-derivative (Block et al., 1983). We therefore exposed cells to inputs where stress increases gradually (Figure 4A).

**Figure 4—figure supplement 1.**
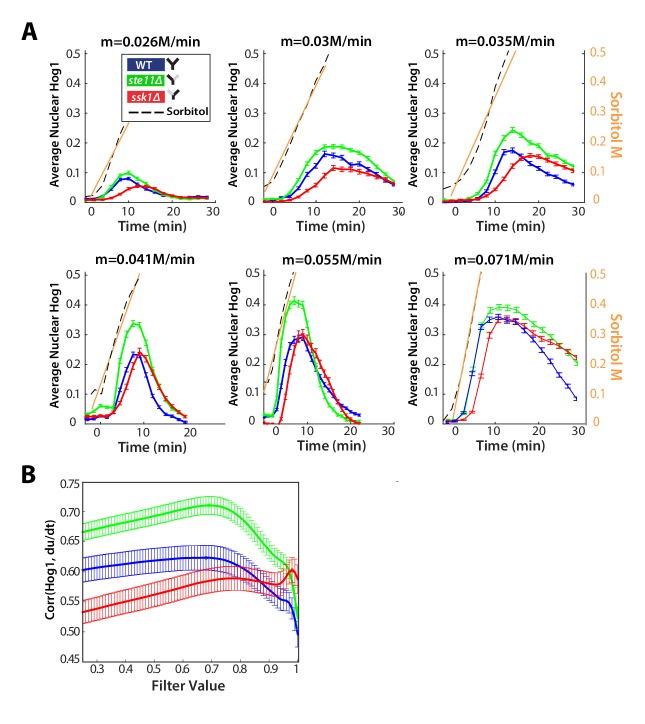
The Hog1 response of the fast mutant in ramps of stress indicates derivative action. (**A**) Ramp data from Figure 2D showing the consistent overshoot of the wild-type Hog1 by the fast mutant. The sorbitol concentration was calculated from the fluorescent signal of the cy5 dye (black dotted lines and right y-axis) and a linear approximation is shown by the orange lines. Numbers of cells are listed in order of wild-type, ste11∆, ssk1∆ for each experiment (n = 201, 195, 192 for 0.026 M/min; n = 112, 198, 97 for 0.03 M/min; n = 193, 226, 200 for 0.035 M/min; n = 175, 164, 144 for 0.041 M/min; n = 134, 86, 60 for 0.055 M/min; n = 148, 187, 186 for 0.071 M/min). (**B**) The cross-correlation between the single-cell trajectories of Hog1 and the time-derivative of the input in the fluctuating ramp of Figure 4D. The derivative was smoothed using a first order filter and the correlation is plotted as a function of the smoothing parameter α. Cross-correlation for three experiments were calculated in total and the average is shown in Figure 4E. **DOI:** http://dx.doi.org/10.7554/eLife.21415.011

In contrast to steps, ramp inputs reveal a substantial phenotypic difference between the fast mutant and the wild-type. We observe a systematic increase in the amplitude of Hog1 in the fast mutant over that of the wild-type and the mutant responds quicker (Figure 4A and B; Figure 4—figure supplement 1). Indeed, the mean wild-type response is closer in amplitude to the slow mutant, particularly during adaptation (Figure 4A). The overshoot of the fast mutant (Figure 4C) is consistent with stronger derivative action in the fast pathway. Nevertheless, Hog1 in the fast mutant adapts despite the ramp in stress, and so the fast pathway must not only have derivative action but also respond to other aspects of the input. Indeed, linearizing a mathematical model of adaptation with negative feedback (Behar et al., 2007) shows that the network performs a time-derivative of the input in parallel with a proportional response.

We note that the wild-type response is generated by interactions between the two pathways and is not an average of their responses (Figure 4A): the maximum amplitude of the wild-type strain increases linearly with the gradient of the ramp although the response of strains with each individual pathway does not (Figure 4C).

To test further the existence of derivative action in the fast pathway, we exposed cells to fluctuating ramps, which have varying time-derivatives (Figure 4D), and determined if the response of Hog1 in each strain best correlated with either the input or the time-derivative of the input. As expected, both the fast mutant and the wild-type have the highest statistical dependency with the (smoothed) time-derivative (Figure 4E and F; Figure 4—figure supplement 1).

The cellular response to osmotic stress should be sufficiently fast to enable survival. Our results are consistent with this task being addressed principally by the fast pathway, which initiates a ‘knee-jerk’, reflex-like response, partly through derivative action, that can overshoot and be too fast in comparison to the wild-type in ramps of stress.

### Interactions between the two pathways enables the wild-type response

Together our results indicate contrasting roles for the two input pathways: the slow pathway provides accuracy at the expense of speed (Figure 3); the fast pathway provides speed at the expense of accuracy (Figure 4). Further, the response to ramps of stress implies the outputs of the two pathways do not always sum to give the wild-type response (Figure 4A).

**Figure 5.**
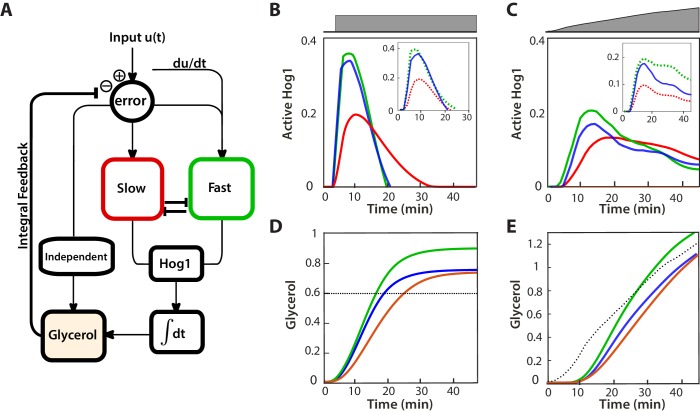
A mathematical model with interactions between the pathways can describe the behaviours of the wild-type and mutants. (**A**) A block diagram of a modular model of the HOG network. The slow pathway responds to the error, the difference between the intracellular and extracellular osmolarity; the fast pathway responds to both the error and the time-derivative of the input u (the extracellular osmolarity). These pathways mutually inhibit each other and then activate Hog1. The rate of change of glycerol is determined by both the time-integral of Hog1 and by the level of activation of a Hog1-independent pathway that responds proportionally to the error. The accumulation of glycerol determines the intracellular osmolarity and the network’s negative feedback. (**B,C**) Predictions of the wild-type and the two mutants in steps (0.6 M) and ramps (0.03 M min^−1^) of stress. The inset shows the contributions of the fast and slow pathways (dotted) to the wild-type response. Mutations remove cross-inhibition between the pathways causing the behaviour of the mutants to be different from the behaviour of the corresponding pathway in the wild-type. (**D,E**) Predictions of glycerol show that all strains initially over- or under-shoot the long-term behaviour (dotted black line). The fast mutants overshoots in both cases. **DOI:** http://dx.doi.org/10.7554/eLife.21415.012 10.7554/eLife.21415.013Figure 5—source data 1.Parameters for the mathematical model of the HOG network.Values for the zero-order gain and time-constants of the different components of the block diagram shown in Figure 5—figure supplement 1.**DOI:** http://dx.doi.org/10.7554/eLife.21415.013

Building on previous work (Mettetal et al., 2008; Muzzey et al., 2009), we developed a modular mathematical model of the network with the aim of highlighting general principles governing how cells might balance two opposing tasks. Control theory is a natural framework to describe the modulation of cellular responses (Yi et al., 2000; El-Samad et al., 2005), and correspondingly we present the model as a block diagram (Figure 5A; Figure 5—figure supplement 1).

**Figure 5—figure supplement 1.**
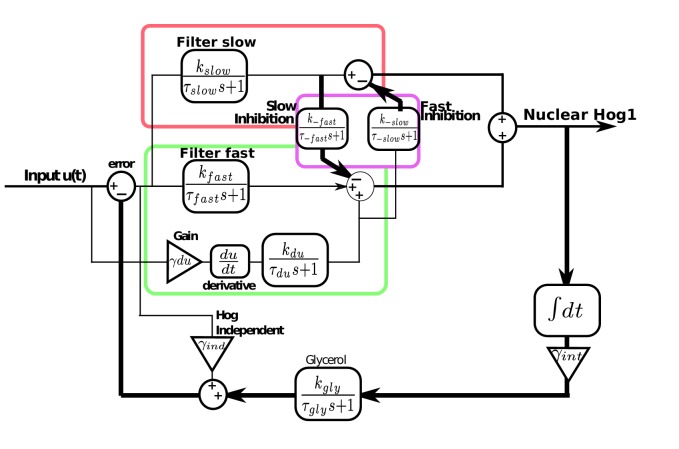
Modular model of the HOG network including derivative action in the fast pathway and the interactions between the fast and slow pathways. The slow pathway is modelled as a first-order filter of the error. The fast pathway also has a first-order filter of the error and derivative action: an amplified and filtered time-derivative of the input. Cross-inhibition between the two pathways consists of negative feedback of the filtered output of the slow pathway on the fast pathway and negative feedback of the filtered derivative action of the fast pathway on the slow pathway. Hog1 is the sum of the outputs of both pathways after the cross-inhibition and feeds into an integrator that is amplified and filtered before feeding into glycerol. The Hog1-independent pathway responds proportional to the error and directly feeds into glycerol. Glycerol negatively feeds back on the input to give the error. All parameter values are Figure 5—figure supplement 1. **DOI:** http://dx.doi.org/10.7554/eLife.21415.014

We modelled the Hog1 response as the output of the cell’s ’controller’ and the production of glycerol as the process being controlled. The integral feedback, which exists because glycerol affects the volume, makes the system a closed loop, and the network as whole acts to reduce the ‘error’—the difference between the intracellular osmolarity (predominately determined by glycerol) and the extracellular osmolarity (the stress or input) (Klipp et al., 2005; Muzzey et al., 2009).

The slow pathway passes the error through a low-pass filter (the time-scale of this filter is determined by the adaptation time of the pathway) and then responds proportionally to the filtered error. Low-pass filtering in this pathway has been observed previously (Hersen et al., 2008).

In contrast, the activation of the fast pathway has two sources: it both responds to the error and to the time-derivative of the input. The error passes through a low-pass filter that has a higher cut-off frequency than the filter for the slow pathway (Hersen et al., 2008). The overall (zero-frequency) gain of the pathway is higher than the gain of the slow pathway, and the response of the pathway is therefore faster.

To generate the wild-type behaviour, the two input pathways inhibit each other (Figure 5A), but the model is agnostic to the biochemical details of this inhibition. A possible mechanism is competition between the pathways for Pbs2 (both pathways activate Pbs2 via phosphorylation of the same two residues [Hohmann, 2002]). For example, each pathway may be able to sequester Pbs2 from the other. This sequestering could potentially arise either from the different spatial locations of the receptors—Sln1 is observed throughout the plasma membrane, but Sho1 localizes to sites of polarized growth (Raitt et al., 2000; Reiser et al., 2000)—or from different allosteric states of Pbs2 (Monod et al., 1965). Indeed, from our data (Figure 3B), the amount of Pbs2 is limiting because the response of Hog1 changes if the levels of Pbs2 are reduced. Nevertheless, the inhibition could also be indirect: for example, the slow pathway could positively feedback on the fast pathway, which in turn inhibits the slow pathway.

The output of this biochemical controller is Hog1, whose activation is determined by the cross-inhibition between the input pathways. Hog1 feeds into an integrator, which determines the levels of glycerol and gives the system integral feedback (Muzzey et al., 2009). This integrator could be a long-lived gene product whose transcription is activated by Hog1 and whose levels are therefore proportional to the total amount of time that Hog1 spends in the nucleus.

Finally, Hog1-independent mechanisms are know to regulate the early accumulation of glycerol (Brewster et al., 1993). For example, the Fps1 channels, which export glycerol through the plasma membrane, are not only controlled by Hog1, but also have additional regulation (Luyten et al., 1995; Ahmadpour et al., 2016). Their fast closure gives an initial boost of glycerol (Petelenz-Kurdziel et al., 2013), which is important in all three mutants because the glycerol produced from nuclear Hog1 accumulates relatively slowly, over the time-scale of the integrator. We include such mechanisms as an additional input pathway that responds proportionally to the error and directly controls glycerol (Muzzey et al., 2009). We note that if the error becomes negative, this pathway reduces intracellular glycerol and therefore partly describes the effects of open Fps1 channels.

The model captures the differences between the mutants and the wild-type we observe in both steps (Figure 5B) and ramps (Figure 5C) and provides insight into how two opposing tasks can be implemented in the network by having specialized subnetworks. Analogous specialization is believed to occur, for example, in the establishment of polarity in yeast where the speeds of activating and de-activating of the relevant signalling network are made distinct by having two separate positive feedbacks (Brandman et al., 2005).

### The architecture of the HOG network enables both speed and accuracy

Using the model, we can understand the architecture of the HOG network as a means to provide both speed and accuracy. A fast response requires a high gain, but increasing gain typically comes with a reduction in structural stability (Astrom and Murray, 2008). Within the model (Figure 5D and E), this instability can manifest as the levels of glycerol overshooting and potentially oscillating (Schaber et al., 2012). Uncontrolled production of glycerol decreases the accuracy of the response by causing a mismatch between the dynamics of Hog1 and the dynamics of the volume.

Derivative action is a well-known way to increase gain while maintaining structural stability (Astrom and Murray, 2008), and derivative action in the fast pathway enables that pathway’s high gain. The derivative action is open loop, responding to the input not the error, and therefore reduces the coupling of the fast pathway to the integral feedback, undermining the network’s accuracy. The intrinsic time-scale of the derivative action in the fast pathway is highlighted in the Pbs2 mutants where a reduction in Pbs2 decreases the gain of only those elements of the fast pathway that are sensitive to the error. Consequently, the derivative action principally determines the adaptation time of Hog1 (Figure 3D). A further signature of the intrinsic time-scale is also present in the non-monotonic character of the mutual information between the adaptation time of Hog1 and the level of stress in both the fast mutant and the wild-type (Figure 2D). This behaviour implies that the derivative action is typically strong enough to dominate the Hog1 response in steps and causes cells to adapt appropriately to the level of shock, at least early in the response.

The presence of the slow pathway allows the network to maintain accuracy despite the open-loop component of the fast pathway. The slow pathway has a lower gain and is therefore slower but more stable (Figure 3B and C). Further, it only responds to the error and can compensate for inaccuracies generated by the fast pathway because it is more sensitive to the integral feedback.

In steps, the wild-type and the fast mutant are initially driven by the derivative action and the quicker response of the fast pathway allows inhibition of the slow pathway. Consequently, the fast pathway dominates the wild-type response (Figure 5B). At later times when the response from the derivative action falls, the differences between the accuracy of the wild-type, which benefits from the additional stabilising effects of the slow pathway, and the fast mutant become apparent. In ramps, the activation of the fast pathway by the derivative of the input is again important giving the overshoot observed in the fast mutant, but the maximum of its contribution is smaller and the initial inhibition of the slow pathway is lower (wild-type Hog1 begins as the average of the two mutants). Once the pathways activate further, they both inhibit each other and both control the response and can reduce the levels of Hog1 in the wild-type below either mutant (Figure 5C).

### Each input pathway favours survival in distinct dynamic environments

The two mutants perform better at different tasks, responding at different speeds and with different levels of accuracy, and if these tasks are important for the cell we expect that the mutations may come with a fitness cost, although potentially only in particular environments. We therefore measured cell viability for stress with three different types of dynamics: steps, linear ramps, and fluctuating ramps.

**Figure 6.**
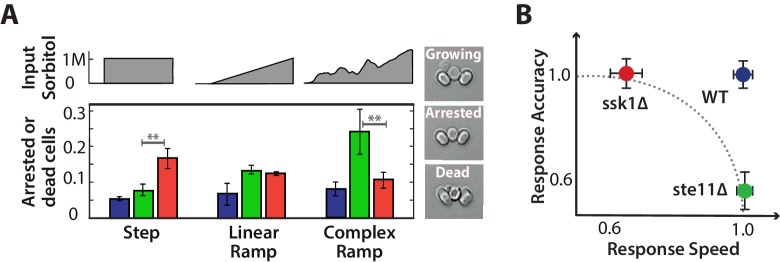
Each input pathway increases survival in specific environments, but the wild-type is the most fit in all environments being both fast and accurate. A We measure failure to grow (inset) by the number of cells that either did not resume the cell cycle (arrested) or die over the 5 hr after the stress has stabilized for three different environments: a 1M step, a 0.03 M min${}^{-1}$ linear ramp (of 40 min length), and 2 hr of a fluctuating ramp. Mean and SD of 2 experiments each comprising at least 300 cells per strain. The asterisks denote significance with a p-value $<10^{-6}$ calculated using a t-test and bootstrapping. See also Videos 2 and 3. B Together our results imply that a network with only one input pathway is subject to a speed-accuracy trade-off, which the two mutants satisfy in contrasting ways and we illustrate by showing the mutants lying on the extremes of a hypothetical Pareto front (dotted line). The wild-type by having two interacting pathways, each specializing to one aspect of the trade-off, escapes this constraint. Mean and 95% confidence intervals for response time and accuracy using data from Figure 2. . **DOI:** http://dx.doi.org/10.7554/eLife.21415.015

Bulk fitness has been previously measured in the two mutants, and growth deficiencies for the slow mutant have been observed at high osmolarity (above approximately 0.3 M salt) (Macia et al., 2009). Such bulk measurements, based on monitoring optical density in liquid media, make varying the environmental dynamics challenging and miss single-cell events. Using ALCATRAS, we measure the number of cells that either die in the stress or never restart growth over five hours once the environment has stabilized. This direct measure allows the performance of individual cells to be evaluated (Figure 6A). All strains grow similarly in rich media and any change in fitness is therefore a consequence of the osmotic stress.

Quantifying the number of unfit cells, we find that the selective advantage of having both input pathways is revealed by exposing cells to environments with a range of different dynamics: the fast mutant outperforms the slow mutant in steps of stress and the slow mutant outperforms the fast mutant in ramps of fluctuating stress (Figure 6A; Videos 2 and 3).

**Video 2.** Survival of wild-type and mutant cells following a step of 1 M sorbitol (related to Figure 6). A representative field of view (DIC channel) showing cells trapped in the ALCATRAS microfluidics device. White arrows indicate cells that are either arrested or dead. **DOI:** http://dx.doi.org/10.7554/eLife.21415.016

**Video 3.** Survival of wild-type and mutant cells following a fluctuating ramp of sorbitol from 0 to 1.2 M over approximately 2 hr (related to Figure 6). A representative field of view (DIC channel) showing cells trapped in the ALCATRAS microfluidics device. White arrows indicate cells that are either arrested or dead. **DOI:** http://dx.doi.org/10.7554/eLife.21415.017

For steps, a fast response is a priority, and although the slow pathway is more accurate, the corresponding greater degree of overshoot of glycerol in the fast pathway, as expected by our model (Figure 5D), does not substantially affect survival of the fast mutant. In the model, the overshoot is counteracted by export through the Fps1 channels, and so the fast mutant pays a greater metabolic cost by exporting more glycerol. This cost does not affect survival but may change other correlates with fitness, such as lag time.

In ramps, where the environment continually changes, the derivative action in the fast pathway does not eventually decrease like it does in steps and the overshoot of glycerol correspondingly has a longer life-time in the fast mutant (Figure 5E) and is therefore likely to be more deleterious. An indication of deleterious effects in higher gradients can be seen in the amplitude of Hog1 (Figure 4C): although the amplitude of the wild-type Hog1 increases linearly with the gradient of the ramp and is therefore likely to be informative about this gradient, the saturating response of the fast mutant implies that its amplitude of Hog1 will often be similar, at least for inputs with higher gradients. Individual cells in the fast mutant may not then be able to match their response to the magnitude of the stress.

In the fluctuating ramps, accuracy is a priority. In the fast mutant, the derivative action depends only on the current value of the input and so the fast mutant responds almost anew each time the stress increases regardless of the cell’s internal state (levels of glycerol). The resulting overshooting of glycerol is therefore compounded because of the long life-times of the overshoots and the ramp’s greater duration. The slow mutant, in contrast, can modulate its response both by the cell’s internal state and by the history of the stress because of its greater sensitivity to the integral feedback. With its two input pathways, the wild-type performs both tasks and is both fast and accurate, always having the highest probability of survival.

## Discussion

We have thus shown that trade-offs in performance can undermine signalling in a single input pathway with either speed being sacrificed for accuracy or vice versa, but that by having two input pathways, each specializing to particular task, signalling networks can mitigate these trade-offs (Figure 6B).

In the HOG network, the Sln1 pathway is a fast reflex-like response that provides the speed necessary to survive sudden shocks but at the expense of accuracy, and alone can cause adaptation of Hog1 before recovery of the cellular volume. Consistent with this observation, Macia *et al.* report that fast mutants have a shorter duration of Hog1 phosphorylation compared to wild-type cells (Macia et al., 2009).

In contrast, the Sho1 pathway provides accuracy at the expense of speed and by specializing to sensing the integral feedback coming from volume recovery is more sensitive to the cell’s internal state and the history of the extracellular stress. This behaviour is consistent with earlier speculations that the Sho1 branch primarily monitors osmotic changes during normal growth (Hohmann, 2002). If the integral feedback is to allow recovery of the volume, the network must remember the cellular volume before the stress (Astrom and Murray, 2008), and the Sho1 pathway interacts with the actin cytoskeleton (Tanaka et al., 2014), which might allow information from cell morphology and growth to be integrated with activation of Hog1.

The two input pathways have been reported to have different thresholds of activation (Macia et al., 2009), but our data and modelling points towards a re-interpretation in terms of different gains for the pathways. For all the steps and ramps of stress that we consider, we observe a response from both mutants, and so any differences in thresholds must be small (less than 0.2 M sorbitol in steps and 0.03 M min${}^{-1}$ in ramps). The advantage of multiple thresholds might be to increase the network’s dynamic range, but given that both thresholds can only be small, such an increase is unlikely to be substantial in the HOG network. Our data points towards it being the interaction between the two pathways that increases the dynamic range: we observe that only the wild-type response increases linearly with the gradient of a ramp of stress (Figure 4C).

A potentially alternative architecture of the HOG signalling network is to have a single fast input pathway controlling the integral feedback. Such a network, however, would not only have structural instabilities in its dynamics because of the high gain necessary for high speed, but also would be more likely to become insensitive to the cell’s internal state for sufficiently high stress. In large stress, all Hog1 molecules can become activated and the output of the HOG controller is then saturated. This saturation will happen for shocks of smaller magnitude for systems with high gain and causes a loss of accuracy because the system is then in open loop and is unable to exploit the integral feedback. Saturated activity of Hog1 should generate maximum production of glycerol and so faster recovery of cellular volume, but, once the volume has recovered, the level of glycerol synthesis will be too high for the level of stress and there will be a fitness cost. Having a slow pathway that inhibits the fast pathway helps prevent saturation of Hog1 activity and so increases sensitivity to the integral feedback for higher levels of stress.

We have developed a block diagram model of the HOG network, but a caveat is that, although the model is therefore modular, it is also agnostic to the biochemical details of both the interaction between the two input pathways and the mechanism allowing the fast pathway to respond to the time-derivative of the input. Competing for Pbs2 is one possible means of cross-inhibition between the pathways, but multiple feedbacks, both positive and negative, exist within the HOG network (Hao et al., 2007; Macia et al., 2009; Sharifian et al., 2015; English et al., 2015), and a feedback-based interaction is possible. That biochemistry can be used to measure a time-derivative on a time-scales as fast as seconds is well established (Block et al., 1983), and, in analogy with bacterial chemotaxis, we expect that upstream signalling in the fast pathway encodes a short-term memory of the level of the input to allow comparison of the current level to a value in the past.

More generally, our results confirm the importance of using inputs with varying dynamics to uncover the logic behind cellular signalling (Nurse, 2008; Alexander et al., 2009). In the wild, organisms are exposed to signals with a wider range of temporal behaviours then the constant inputs typically studied in the laboratory (López-Maury et al., 2008), and signal transduction is likely to have evolved to allow organisms to differentiate between such signals or at least between classes of signals (Bowsher and Swain, 2014; Tkačik and Bialek, 2016). Although such work is still in its infancy, dynamic inputs have been successfully used to understand signalling responses in, for example, bacteria (Young et al., 2013), yeast (Hao et al., 2013), and mammalian cells (Kellogg and Tay, 2015), and, with the ease of use of microfluidics (Bennett and Hasty, 2009), we believe should become commonplace.

In conclusion, we have shown that cellular signalling is vulnerable to fundamental trade-offs in performance, but that these vulnerabilities can be overcome by distributing tasks to different parts of the network and integrating together the outputs of this division of labour. We therefore expect such improvements in performance by the specialization of subnetworks to different tasks to exist broadly within cellular signal transduction.

## Methods and materials

### Strains used

All strains (Table 1) were constructed using PCR-based genomic integration and were validated by colony PCR. For inducible expression of PBS2, we used the Tet-off system (Bellí et al., 1998), for which doxycycline causes repression. We PCR-amplified the kanMX4-tTA-PtetO7 from plasmid pCM225 (Bellí et al., 1998) and inserted to substitute 200 bp upstream of the PBS2 ORF. Correct insertion was verified by colony PCR. The mutants showed equivalent growth from wild-type strains in XY media with 2% glucose over 24 hr (data not shown).

**Table 1.** Saccharomyces cerevisiae strains used. All strains are in the S288C background. **DOI:** http://dx.doi.org/10.7554/eLife.21415.018

Strain	Genotype	Source
BY4741	MATa his3Δ1 leu2Δ0 met15Δ0 ura3Δ0	EUROSCARF
BY4742	MATα his3Δ1 leu2Δ0 lys2Δ0 ura3Δ0	EUROSCARF
SL364	MATa, leu2Δ0, lys2Δ0, HOG1-GFP::HIS3, HTB2-mCherry::URA3	gift - P. Hersen
SL373	MATa, met15Δ0, HOG1-GFP-HIS3, HTB2-mCherry::URA3, ste11::LEU2	gift - P. Hersen
SL268	MATa, leu2Δ0, lys2Δ0, HOG1-GFP-HIS3, HTB2-mCherry::URA3, ssk1::KANMX6	This study
SL395	MATa, leu2Δ0, lys2Δ0, HOG1-GFP::HIS3, HTB2-mCherry::URA3, P PBS2Δ::KANMX4-tTA-P tetO7	This study
SL396	MATa, met15Δ0, HOG1-GFP::HIS3, HTB2-mCherry::URA3, P PBS2Δ::KANMX4-tTA-P tetO7, ste11::LEU2	This study
SL442	MATa, lys2Δ0, HOG1-GFP-HIS3, HTB2-mCherry-URA3, P PBS2Δ::kanMX4-tTA-P tetO7, ssk1::Hph	This study

### Microscopy and microfluidics

#### Cell preparation and loading ALCATRAS

Cells were grown at 30°C overnight in 2% glucose in synthetic complete (SC) media, diluted by a factor of 1/20 into fresh SC with 2% glucose next morning and incubated for 4 hr at 30°C. Cells were then loaded into the ALCATRAS chamber (Crane et al., 2014), which was already filled with SC with 2% glucose and 0.05% bovine serum albumin (BSA) to facilitate cell loading and reduce the formation of clumps of cells. Cells were allowed to rest for 1 hr in the microfluidic chamber before stress was applied.

The microfluidic chamber and syringe pumps were located inside an incubation chamber (Okolabs) that maintained a constant temperature of 30°C. We used a 60 × 1.4 NA oil immersion objective (Nikon). To ensure consistent focus over the experiment, the Nikon Perfect Focus System (PFS) was used. Fluorescence imaging was performed with an OptoLED light source (Cairn Research). Images were acquired using an Evolve 512 EMCCD (Photometrics).

#### Dynamically changing extracellular stress

Two syringe pumps (Aladdin NE-1002X) were used to create dynamic environmental conditions. The first pump was loaded with SC and 2% glucose; the second pump was loaded with SC with 2% glucose and sorbitol. Both pumps infused media into a sterile metal T-junction in which media was mixed and then dispensed into the microfluidic chamber. We control the ratio of the two media entering the microfluidic chamber by setting the relative flow of each syringe pump. The total flow rate into the chamber was 4 μL/min for all experiments.

The dye Cy5 was added to the syringe containing sorbitol and used to monitor the sorbitol dynamics. The concentration of sorbitol we report corresponds to the level of fluorescence from Cy5.

#### Multi-strain experiments

To expose multiple strains to the same environmental conditions and to optimize data acquisition, we developed a multi-chamber version of ALCATRAS, which allows different strains to be loaded into distinct chambers but still be exposed to the same extracellular media. Polydimethylsiloxane (PDMS) barriers between strains ensure that there is no cross-contamination during loading. We correct for a delay of few seconds in image acquisition as the microscope moves between different positions.

#### Image segmentation and quantification

During each experiment, we acquired both DIC and fluorescence images. In the z-direction, fluorescence images were acquired in 0.75 micron steps over a range of six microns. The maximum projection of these images (the maximum pixel values across all the images) was used for quantification. In contrast, the Cy5 channel used to quantify the level of sorbitol in the media was acquired at a single focal plane.

Cells were segmented using the DIC images and custom Matlab code that used a support vector machine classifier to identify the centres of the cells. The size of each cell was first estimated with the circular Hough transform and then refined using an active contour method applied to the fluorescence images (Bakker et al., 2017; Bakker and Crane, 2017). A copy of the cell segmentation software is archived at https://github.com/elifesciences-publications/segmentation-software.

The volume of each cell was approximated from the total area of the cell in the fluorescence image (Gordon et al., 2007; Muzzey et al., 2009). We calculated the radius of the circle that has the same area as the cross-sectional area of the cell and used this radius to compute the volume assuming spherical cells. Our cell population is mostly young (as an inevitable result of exponential growth), and the majority of cells are approximately spherical.

#### Quantifying nuclear accumulation

We quantified the nuclear accumulation of Hog1 by calculating the ratio between the average of the five brightest pixels within the cell and the median fluorescence of the whole cell. This measure is robust to bleaching of the fluorophore and has been widely used (Cai et al., 2008; Hao and O'Shea, 2011; Lin et al., 2015). Nevertheless, using a strain in which a histone was tagged with mCherry (Htb2), we validated the approach and observed no significant difference between the direct and indirect measures in agreement with an independent assessment (Uhlendorf et al., 2011). Furthermore, using a reporter for the nucleus creates a delay of a few seconds between acquiring the Hog1-GFP images and the mCherry nuclear tag, which can introduce errors when the cells shift between acquisitions, particularly during osmotic shocks.

#### Numbers of cells

With our multi-strain experimental set-up, we can acquire on average 150 cells per strain with a sampling rate of 2 min. Therefore, experiments involving fluorescence imaging usually comprise 150 cells. Experiments with no fluorescence imaging, however, such as the fitness assays in Figure 6, comprise at least 300 cells per strain per condition (using a sampling rate of 5 min). Biological repeats (identical experimental conditions applied to different cultures of the same strain) were performed on different days.

#### Comparing the Hog1 response of different strains

We did observe a dependence of the Hog1 response with the size of the cell, perhaps because of differences in age or pre-culture despite being treated identically. To control for these outliers, we used the unstressed wild-type cells for each experiment to determine a range of valid sizes: between the 20’th and 80’th wild-type percentiles. We excluded cells outside this range when visualizing Hog1.

#### Response times

Single-cell time traces of Hog1 and volume were normalised by the pre-stimulation level (mean of the three time points prior to the shock). For accurate estimation of the response time, we interpolated the Hog1 traces of single cells. The Hog1 trajectories were then re-scaled such that the pre-shock level equals 0 and the extreme value equals 1. The response time distributions (Figure 4B) were computed from pooled data comprising multiple experiments (total cells: wild-type, 772; fast mutant, 833; slow mutant, 626; Figure 4—figure supplement 1). For Figure 6B, we used 1 min sampling data for 0.6 M, 0.8 M and 1 M shocks (two repeats per experiment; total cells: wild-type, 458; fast mutant, 468; slow mutant, 350).

#### Correlation of adaptation time of Hog1 and the recovery time of the volume

To calculate the adaptation times of Hog1 and cellular volume we smoothed the single-cell data using a moving average window (three time-points using smooth in Matlab). Hog1 and volume trajectories were re-scaled such that the pre-shock level equals 0 and the extreme value (either the maximum of the Hog1 response or the minimum of the cell volume) equals 1. Adaptation times to a given percentage of recovery were estimated from the scaled trajectories using linear interpolation.

To find the correlation between the adaptation time of Hog1 and the time of volume recovery (Figure 2C), we pooled data from six step experiments (Figure 2—figure supplement 1) and used bootstrapping to estimate the 95% confidence intervals for the correlation coefficient (bootci in Matlab). Our estimates are based on 2000 bootstrap samples and data points were weighted by experiment to correct for differing numbers of cells. Figure 3—figure supplement 1C was calculated similarly from the data shown in Figure 3—figure supplement 1.

In Figure 2D, we estimated the mutual information between the adaptation times of Hog1, $t_{\mathrm{Hog1}}$, and the magnitude of the stress, u, using MI(t;u)=H(t)-H(t|u). The first term, the entropy of the marginal distribution of adaptation times, was estimated by pooling data from the four step experiments with the highest stresses (Figure 2—figure supplement 1). The distribution of adaptation times for low shocks have higher measurement errors because of the fast adaptation. The second term, the conditional entropy, was obtained by estimating the entropy of the distribution of adaption times for each step experiment and forming their weighted average based on the number of cells in each experiment. Estimates and error bars for all entropy terms were obtained using a Bayesian method (Nemenman et al., 2004) and shown as posterior means and standard deviations.

#### Correlation and mutual information for the derivative of the input

The time-derivative of the fluctuating ramp input was filtered (using filter in Matlab) with the following discrete-time transfer function: H⁢(z)=1/(1-α/z). Trajectories, for both Hog1 and the input, were normalized to have a zero time-average, and the filtered derivative of the input was then cross-correlated with the single-cell Hog1 responses (using xcorr in Matlab). The maximum cross-correlation (across lags) was found for different values of the filter parameter α (Figure 4—figure supplement 1), and we show the highest correlation for all α in Figure 4E.

For the estimation of the mutual information between the level of Hog1 and the time-derivative of the input (Figure 4F), an empirical distribution of input derivatives was obtained from the input trajectory of Figure 4D using 30 bins and ignoring negative values of the derivative. Assuming each time-point to be independent, the mutual information between the input derivatives and the levels of Hog1 was estimated for various lags (from 0 to 5 time-points) and the maximum value across lags is reported in Figure 4F.

#### Fitness measurements

Cells were grown in the same conditions as above. Since the experiments were substantially longer, DIC images were acquired to avoid possible stress to the cells and fluorescence was used only to monitor the dynamics of sorbitol. The stress was applied as above, and we then identified cells that either did not resume the cell cycle or died over a window of 5 hr after the environment stabilized.

### Perturbation of Pbs2 expression

#### Design of the Tet-inducible PBS2 system

To control the levels of Pbs2, we used the Tet-off system (Bellí et al., 1998), for which doxycycline causes repression. The gene of interest is regulated by the Tet transactivator tTA, which consists of the tetO7-binding moiety plus the VP16 activator moiety and is unable to bind DNA when bound by doxycycline.

#### Levels of doxycycline used and growth conditions

For each strain, we chose the concentration that affected the Hog1 amplitude in approximately the same fashion: the three strains respond differently to doxycycline (DOX). We defined three levels of Pbs2: (i) full induction (0 μg/mL DOX for all strains); (ii) medium repression (Hog1 amplitude falls within 50–70% compared to full induction: 0.05 μg/mL for wild-type; 0.03 μg/mL for ste11Δ; and 0.01 μg/mL for ssk1Δ); (iii) high repression (Hog1 amplitude is 20–40% of that in full induction: 0.1 μg/mL for wild-type; 0.1μ g/mL for ste11Δ; 0.06 ug/mL for ssk1Δ).

For each experiment, cells were grown for 4 hr in XY media with 2% glucose then diluted 1/100 into SC with 2% glucose and the indicated amount of doxycycline for overnight growth at 30°C so that Pbs2 reached steady-state levels of expression (cells were under constant selection).

#### Hog1 and volume adaptation with decreased Pbs2

For comparing adaptation times of Hog1 and volume in single cells, we calculated the adaptation time of Hog1 from the time at which the nuclear level of Hog1 accumulation is maximum to the time of 85% recovery of the pre-shock state. The adaptation time of the volume was calculated equivalently, but using the minimum instead of the maximum value reached by the volume. We performed three independent experiments for each of the levels of PBS2 induction.

### A mathematical model for the wild-type and mutants

To model the HOG network we adopted a modular approach based on control theory (Astrom and Murray, 2008) and used Matlab’s modelling platform Simulink. The architecture of the model is shown in Figure 5—figure supplement 1.

Building on previous studies (Muzzey et al., 2009), we developed a linear model in which the different components of the system are first-order linear time-invariant systems characterized by a transfer function $\frac{k}{\tau \,s+1}$ with a zero-frequency gain of k and a time-constant of τ. The error is defined as the difference between the external input, u⁢(t) (the extracellular osmolarity), and the internal state, g (the intracellular concentration of glycerol). The slow pathway responds to the error; the fast pathway responds to the error and additionally has a derivative component that depends directly on the input u⁢(t). The two pathways inhibit each other at different time-scales: $\tau_{\mathrm{fast}}$ and $\tau_{\mathrm{slow}}$. The output of the two pathways, after the cross-inhibition, is added to generate the wild-type response of Hog1, which affects levels of glycerol through an integrator. Finally, the Hog1-independent pathway responds proportionally to the error and feeds directly into glycerol. To simulate the mutants, we remove one input pathway and the cross-inhibition between the pathways.

To parameterize the model, we used parameter optimization in the Simulink platform to simultaneously fit the response of the mutants to a step (0.6 M) and a ramp (0.03 M min${}^{-1}$). We then incorporated the cross-inhibition between the two pathways and fitted the inhibition parameters, $\tau_{\mathrm{fast}}$ and $\tau_{\mathrm{slow}}$, to the wild-type response.

A Simulink file is available as supplemental material.

### Data availability

Data shown in the figures is freely accessible at dx.doi.org/10.7488/ds/2043.
