# Supplementary material for: Distributing tasks via multiple input pathways increases cellular survival in stress
Source: eLife. 2017 May 17;6:e21415. doi: 10.7554/eLife.21415 (PMC5464774; doi:10.7554/eLife.21415)
Supplement: Related texts

# Article commentary: Decision letter

**DOI:** 10.7554/eLife.21415.019

**Affiliations:** Weizmann Institute of Science, Israel

In the interests of transparency, eLife includes the editorial decision letter and accompanying author responses. A lightly edited version of the letter sent to the authors after peer review is shown, indicating the most substantive concerns; minor comments are not usually included.

Thank you for submitting your article "Multiple input pathways improve perception in a MAP kinase network by enabling distributed tasks" for consideration by *eLife*. Your article has been reviewed by three peer reviewers, and the evaluation has been overseen by Naama Barkai as the Senior Editor and Reviewing Editor. The following individual involved in review of your submission has agreed to reveal his identity: Stefan Hohmann (Reviewer #2).

The reviewers have discussed the reviews with one another and the Reviewing Editor has drafted this decision to help you prepare a revised submission.

Summary:

The authors use single-cell analysis to study the role of the two branches of the high osmolarity response in budding yeast. Following results of careful experiments, they suggest differential roles for the two branches: the Sln1 branch is fast and inaccurate and the Sho1 branch is slow but accurate. All reviewers found the work interesting and important.

Essential revisions:

1) Improve the writing. In particular, discuss the relation of the study with previous theoretical study (Brandman et al). In addition, the logical flow and rational for the different should be better described, so that the conclusions leading to the final model is easier to understand. Alternative explanations (e.g. extending dynamics range) should also be mentioned and discussed.

2) Test a Pbs2 over-expression strain as a way to test the assumed key role of competition between the branches for limited Pbs2.

3) Relate to the possible discrepancies mentioned in the reviews. This includes assumptions used in the model that may contrast data from literature, and possible inconsistencies between figures.

Reviewer #1:

"Multiple input pathways improve perception in a MAP kinase network by enabling distributed tasks" by Granados et al. explores the role of the two branches of the high osmolarity response in budding yeast. The authors provide evidence and suggest a model that support differential roles for the two branches of the pathway – the Sln1 branch is fast and inaccurate and the Sho1 branch is slow but accurate. They perform a number of careful single cell observations in well-controlled dynamic environments to support these claims and then extend the work by show the ramification of these effects in physiologically plausible environments. On the whole I enjoyed this work and believe it is a worthwhile contribution towards understanding the design of signaling networks and the emergent properties that can result for certain network designs. I have several points that I would be interested in seeing the authors address.

1) When I first read the Abstract of this paper I thought the authors were going to extend from the work of Brandman et al. (Science 2005). The claims in this paper are similar to the theoretical argument put forth by Brandman et al. for the potential behavior of interlinked negative feedback loops. While not emphasized, a critical feature for the Brandman paper was the need for saturation between the fast and noisy branch and the slow and accurate branch. This paper is a nice advance in that it provides a model and evidence for a practical implementation of this constraint. The authors should evaluate and discuss their contribution in relation to this paper.

2) The authors focus on the kinetics and accuracy of the two responses. Maybe, naively, it seems to me that an equally plausible explanation for the results is that the Sln1 branch responds to large deviations, while the Sho1 branch responds to small deviations – the point of the two pathways is to extend the range of concentrations over which the pathway can work. I would like to see the authors address this alternate possibility and if possible provide evidence against this alternative possibility.

3) Experimentally, it seems like the authors should be able to overexpress Pbs2. Overexpression should be able to eliminate the competition between the two branches of the pathway and the WT response would now become the linear addition of the two pathways.

4) While out of scope of this paper, it would be interesting to see what kinase dead versions of each branch of the pathway did to the overall response.

Reviewer #2:

This single cell analysis of yeast HOG pathway osmostress signalling addresses the role and collaboration of the two branches that sense osmostress and signal to the MAPKK Pbs2 and eventually the MAPK Hog1. The two branches are controlled, respectively, by the Sln1 phosphorelay system and the Hkr1-Msb2-Sho1 complex. The authors define the different branches as fast but inaccurate and slow but accurate and they provide data from well-designed microfluidic experiments as well as simulations from mathematical modelling that support this notion.

The study seems to be well performed, both experimentally and in terms of mathematical modelling. Data analysis also seems to be well developed and appropriate. The results seem to be well documented. My comments mainly concern some conceptional considerations that at least should be discussed.

The role of the two branches has been studied previously and those papers are cited. The present study adds novel information, especially with respect to the above-mentioned idea of different tasks for quick responses to acute stress versus more accurate responses to gradual changes of external osmolarity.

It seems that the authors do not mention that it has been shown that the two branches seem to have different threshold levels for osmostress.

The study suffers from the same limitations as previous studies. One of those refers to the fact that the two branches are studied in isolation, i.e. when the other branch is inactivated by mutation. Probably the two branches behave differently in such a situation. This notion is supported by the data presented here. It is still not known how the two branches operate in wild type cells because reagents to monitor Pbs2 phosphorylation/activity do not exist; it appears in fact that the Sho1 branch contributes little if anything, at least under the scenarios so far studied. This is addressed by the authors in their ramp scenario as well as their mathematical model. But the relevance of the conclusions to the actual wild type situation remains elusive.

Another limitation concerns the fact that the activity of the Slt2 pathway, which responds to opposing osmotic stress treatments and may modulate intracellular glycerol levels, is ignored in the present study (see Talemi et al. 2016). While the HOG and Slt2 pathway do not seem to directly communicate they may do so via control of glycerol accumulation (see for instance Ahmadpour et al. 2016, Baltanas et al. 2013); the Slt2 pathway hence may affect the behaviour of HOG and especially the two branches when they operate in isolation.

The study is also based on some assumptions that do not seem to be supported by data reported in the literature. For instance (Introduction, seventh paragraph) it has been reported that HOG feedback coincides with the onset of volume recovery, i.e. as soon as the cells start to recover volume, the HOG signal falls rapidly back to basal. This implies, that the sensors sense changes in volume rather than absolute volume. See for instance Babazadeh et al. 2013.

Results, first paragraph: there are additional single cell studies, some are cited elsewhere, some not: Schaber et al. 2012, Babazadeh et al. 2013, Sharifian et al. 2015 to name a few.

It appears that the authors do not explicitly mention that they chose sorbitol as osmostress agent. Most previous studies employed NaCl. The authors should justify their chose for the agent and the concentrations employed.

Reviewer #3:

The paper by Granados et al. fundamentally asks the question of how the two branches of the osmo-stress pathways coordinate to produce a rapid, yet accurate, response. The paper presents intriguing data that argues for temporal "hand-over control" from the fast (Sln1/Ssk1) branch to the slow (sho1) branch. As mentioned, the Sln1/Ssk1 is fast and dominates the early response through a bigger share of a common resource of the pathway (PBS2) and when this fast response weans away, PBS2 is liberated for the second branch, the slow one which is now handed the reins. Evidently, the presence of integral control in the slow branch is instrumental to this story, given that it is responsible for matching the perceived stress to the actual stress. As a result, this slow branch might be the major driver of response "accuracy", a hypothesis supported by some data in this manuscript.

I will jump to the bottom line here: Above is the story that I extracted from the paper after reading it multiple times, trying to circumvent distractions to this narrative such as inter-spaced descriptions of step and ramp responses, and also a meager description of the computational modeling, which in my opinion should play a more prominent role in explaining the data. The data is not intuitively obvious, and the paper as written leaves the conclusion to the imagination of the reader. The narrative is ridden with jargon ("accuracy" is such a term), and crucial primary data is not shown (the volume time trajectories, rather a correlation plot between data features that the reader never sees is shown in a major figure that is actually the most important one of this work). There is obviously important information in these data not explicitly shown, since the correlation between volume recovery and Hog1 response seems to be changing with time and the Hog1 response shown for the ssk1 mutant has a rather intriguing biphasic recovery shape at high salt concentration (Figure 3 and accompanying supplementary figures). In light of these and other confusing elements, I found myself entranced by the narrative and model proposed by the authors, but confused as to 1) whether I am interpreting it correcting, or projecting my own partial understanding), 2) if I am understanding it correctly, then the data presented and explanations provided only partially corroborate this model and 3) I truly didn't know what to do with the ramp input data, was it there to make a point that supports the model or to argue that different branches of the pathway are instrumental in different conditions?. So, my rather unorthodox recommendations are:

1) Rewrite the paper to focus on a clear narrative. What is exactly the model that the authors are proposing? Present that *clearly* (including why the computational model explains the data), and present the data in the order and logical sequence that actually support this model.

2) Present all the data for volume recovery, the same as presenting the data for the Hog1 nuclear residence, not summary statistics of it.

3) Bring the computational modeling to the forefront. For example, if the model I extracted out of the paper is correct, then I don't intuitively understand why the Ste11 mutant would have an identical Hog1 response to WT. I would have expected it to have the same initial response, maybe the same or similar Hog1 peak, but then the adaptation phase to be different. Why is it not? I am sure there is an explanation, but it is nowhere to be found in the manuscript. This is not a superficial point, this goes to 1) assessing the rigor and robustness of conclusions and 2) actually giving the reader information that they understand, and therefore trust.

Suggestions above only give a partial list of changes that are needed. I intended to write those as a motivation for the authors to take a deeper look at their very intriguing and beautifully collected data, and extract the most robust story out of it.

A few other concrete questions:

1) In Figure 2A, the fast activation Hog1 localization is virtually indistinguishable from the WT; however, in Figure 3A, the fast mutant loses accuracy as the recovery continues. Since the fast mutant's nuclear import of Hog1 is virtually identical to the WT, and synthesis of Gpd1 and Gpp1 has been demonstrated to be the single most important factor towards recovery (Babazadeh et al., 2014), one would expect the resulting glycerol synthesis to be similar, but this argues differently. At what point in the stress perception does this "knee-jerk" reaction fail the cell? The authors use the recovery in volume as a proxy for actual stress recovery, but this is the secondary effect from Hog1's interaction with its binding partners and glyercol production. In addition to the change in volume, I would like to see a representative promoter's activity (e.g. pSTL1-YFP) to directly observe where the breakdown in cellular perception occurs. Given the argument that the slow mutant gains in accuracy over time, I would expect to see different promoter dynamics in the two mutants in their optimal environments. This could substantiate the author's claim of the two roles the pathways play.

2) In Figure 5 (and Figure 5—figure supplement 2), the authors demonstrate an osmostress terrain that should favor the pathway that is able to integrate better – and subsequently demonstrate the "fast" pathway's disadvantage. How were these terrains chosen? Both ramps loosely resemble (and therefore could be interpreted as) ramps, and it's surprising to find the "fast" mutant at nearly a 2X disadvantage (Figure 6A). This may be something more appropriate for the supplemental, but it would be nice to see a more examples of "complex" environments that are more distinct from a classical incline ramp to really cement the accuracy vs. speed argument. What are the corresponding volume (and promoter?) dynamics, and how to they correlate with the Hog1 localization shown in Figure 5D?

3) Mitchell et al. (2015) touched upon how various waveforms are interpreted by the Hog1 pathway (i.e. high frequency is a single step; moderate frequency is a staircase; and low frequency is moderate staircase). This seems very relevant to the model the authors propose; How does the model proposed in Mitchell et al. and their work reconcile?

4) In the Discussion, the authors state "…this 'passing on' of control occurs predominately through competition for Pbs2." This is a crucial aspect of the model proposed. While this was explicitly modeled, and the resulting Hog1 dynamics matched the predictions, the sharing model of Pbs2 was never experimentally tested. The authors propose that the different spatial localizations of Sho1 and Sln1 could aid in sequestering Pbs2. Since Sho1 localizes to the region of polarized growth, they should tag Pbs2 and Sho1 and observe co-localization at the onset of osmostress. If not possible within a reasonable timeframe, then the authors should spend some time providing evidence from literature supporting this aspect, or if none exists, state that explicitly in order to set the record straight.

# Reply: Author response

**DOI:** 10.7554/eLife.21415.020

Essential revisions:

1) Improve the writing. In particular, discuss the relation of the study with previous theoretical study (Brandman et al). In addition, the logical flow and rational for the different should be better described, so that the conclusions leading to the final model is easier to understand. Alternative explanations (e.g. extending dynamics range) should also be mentioned and discussed.

We have completely restructured the paper in order to both incorporate the comments from the reviewers and clarify the narrative. All the data for steps of stress inputs are now discussed together and before we introduce the study of ramp inputs. We have clarified and simplified our definition of accuracy and developed a new, more intuitive mathematical model of the system that helps provide a clear take-home message.

We now include the Brandman et al. paper in the section “Interactions between the two pathways enables the wild-type response”. Although there are important similarities with our findings, particularly that the two tasks of switching on and switching off that they consider can be distributed to separate parts of a signal transduction network, there are important differences too. Brandman et al. consider switching-off times, which are equivalent to the deactivation time of Hog1 when stress is removed. These deactivation times are known to be the same for the two inputs pathways in the HOG network (Hersen et al., Proc Nat Acad Sci USA 2008) and are not equal to the adaptation time in the continual presence of stress that we consider. Also Brandman et al. focus entirely on positive feedback. In our framework, the two input pathways negatively feedback on each other, and, although negative feedback by component A on a component B that itself negatively feeds back on component A is positive feedback, we do not feel the connection to Brandman et al. in this respect to be strong. Finally, Brandman et al. consider robustness to fluctuations in the input whereas we consider structural instabilities in the glycerol response, which although they may be enhanced by stochastic effects are fundamentally different because such instabilities remain in deterministic models.

2) Test a Pbs2 over-expression strain as a way to test the assumed key role of competition between the branches for limited Pbs2.

This is an excellent suggestion to verify the potential importance of the competition for Pbs2.

We did over-express PBS2 using a DOX inducible system, but, in spite of considerable effort (which delayed our resubmission), we were unable to obtain consistent results. Cells incurred strong fitness costs and there was a high degree of heterogeneity in cell responses. As such, these results are not of a sufficient standard to be published. In the literature, over-expression of PBS2 is indeed known to reduce fitness and a complete growth arrest has been reported with high over-expression (Krantz et al., Mol Syst Biol 2009). To reduce these deleterious effects, other groups have transiently over-expressed PBS2 in diploid cells where one copy of PBS2 is wild-type and only the second is inducible (Dexter et al., BMC Syst Biol 2015). Our study, like the vast majority, is entirely haploid-based, and we believe that comparing haploid with diploids may lead to erroneous conclusions.

We understood by suggesting over-expression of PBS2 that the underlying concern of the reviewers is that our model is too specific given our data. To counter this concern, we have developed a new control theory-based model of the HOG network that recapitulates our experimental results, is more intuitive, and does not rely on any specific molecular mechanism (although it is of course consistent with known biochemistry). We believe that this model substantially improves the manuscript and alleviates the requirement for the PBS2 experiment.

3) Relate to the possible discrepancies mentioned in the reviews. This includes assumptions used in the model that may contrast data from literature, and possible inconsistencies between figures.

We believe that there are no discrepancies now present in the manuscript and that the new model ties together well all the data, as well as building on and being consistent with the earlier modelling work of the Van Oudenaarden laboratory (Muzzey et al., Cell 2009).

Reviewer #1:

[…] 1) When I first read the Abstract of this paper I thought the authors were going to extend from the work of Brandman et al. (Science 2005). The claims in this paper are similar to the theoretical argument put forth by Brandman et al. for the potential behavior of interlinked negative feedback loops. While not emphasized, a critical feature for the Brandman paper was the need for saturation between the fast and noisy branch and the slow and accurate branch. This paper is a nice advance in that it provides a model and evidence for a practical implementation of this constraint. The authors should evaluate and discuss their contribution in relation to this paper.

Although we agree there are similarities with Brandman et al., there are important differences too (see our response in Essential revisions above). We now include the Brandman et al. paper in the section “Interactions between the two pathways enables the wild-type response”.

2) The authors focus on the kinetics and accuracy of the two responses. Maybe, naively, it seems to me that an equally plausible explanation for the results is that the Sln1 branch responds to large deviations, while the Sho1 branch responds to small deviations – the point of the two pathways is to extend the range of concentrations over which the pathway can work. I would like to see the authors address this alternate possibility and if possible provide evidence against this alternative possibility.

In our data, which includes shocks and ramp gradients including those of low magnitude, we see that both the fast and the slow mutant always respond (Figure 2—figure supplement 1; Figure 4—figure supplement 1). Our ramp experiments, in particular, demonstrate that both input pathways are sensitive to small deviations. If the pathways do have a threshold of response then these thresholds must be small for both pathways and appear unlikely to substantially increase the dynamic range of the HOG network. Nevertheless, Macia et al., Sci Signal 2009, do report the response to a step of even smaller magnitude to which the slow mutant does not respond, but we believe that this lack of a response can be understood as a consequence of the delay in the slow pathway. During the time that the slow pathway takes to respond, faster Hog1- independent mechanisms alleviate the stress experienced by the cell and so the input to the slow pathway decreases preventing a response. If the two input pathways have different gains, however, then our model can explain our data.

The work we are most familiar with that shows the advantages of having two sensing systems with different thresholds of activation is Levy et al., Science 2011, but there the two thresholds allow cells to predict the direction of change of an input rather than increase the dynamic range. Indeed, negative feedback appears to be a conserved mechanism to increase dynamic range (Madar et al., BMC Syst Biol 2011; Voliotis et al., Proc Nat Acad Sci USA 2014).

3) Experimentally, it seems like the authors should be able to overexpress Pbs2. Overexpression should be able to eliminate the competition between the two branches of the pathway and the WT response would now become the linear addition of the two pathways.

We agree that over-expression should provide insight into the mechanisms by which Pbs2 integrates the signals from the two input pathways. As described in Essential revisions, we did over-express Pbs2 using a DOX inducible promoter but ran into the well-documented deleterious effects of high Pbs2 levels. Instead, we have developed a new model which is agnostic to the mechanistic details of how the two input pathways interact and we believe alleviates the need for the Pbs2 experiment.

4) While out of scope of this paper, it would be interesting to see what kinase dead versions of each branch of the pathway did to the overall response.

Reviewer #2:

[…] It seems that the authors do not mention that it has been shown that the two branches seem to have different threshold levels for osmostress.

We have corrected this oversight and have added some new material to the Discussion, but note that we always see a response from both pathways for all stress (ramps and steps) that we apply and that the previously observed difference in activation thresholds might result from the different gains of each pathway.

Reviewer 1 had a similar question and we repeat our answer here for convenience: In our data, which includes shocks and ramp gradients including those of low magnitude, we see that both the fast and the slow mutant always respond (Figure 2—figure supplement 1; Figure 4—figure supplement 1). Our ramp experiments, in particular, demonstrate that both input pathways are sensitive to small deviations. If the pathways do have a threshold of response then these thresholds must be small for both pathways and appear unlikely to substantially increase the dynamic range of the HOG network. Nevertheless, Macia et al., Sci Signal 2009, do report the response to a step of even smaller magnitude to which the slow mutant does not respond, but we believe that this lack of a response can be understood as a consequence of the delay in the slow pathway. During the time that the slow pathway takes to respond, faster Hog1-independent mechanisms alleviate the stress experienced by the cell and so the input to the slow pathway decreases preventing a response. If the two input pathways have different gains, however, then our model can explain our data.

The study suffers from the same limitations as previous studies. One of those refers to the fact that the two branches are studied in isolation, i.e. when the other branch is inactivated by mutation. Probably the two branches behave differently in such a situation. This notion is supported by the data presented here. It is still not known how the two branches operate in wild type cells because reagents to monitor Pbs2 phosphorylation/activity do not exist; it appears in fact that the Sho1 branch contributes little if anything, at least under the scenarios so far studied. This is addressed by the authors in their ramp scenario as well as their mathematical model. But the relevance of the conclusions to the actual wild type situation remains elusive.

In our opinion this criticism is a little harsh: much of cell and molecular biology were developed with the same approach of using mutations to understand wild-type behaviour.

Nevertheless, we agree that a potential limitation of our work is that we studied the input pathways using mutation and that their behaviour in the wild-type might be different from that in the mutants. As the reviewer suggested, our mathematical modelling is a way to circumvent this criticism because the model is required to fit both the wild-type and the two mutants. In doing so, we in fact predict that the two pathways do behave differently in the wild-type because of the cross-inhibition present in the wild-type and absent from the mutants (Figure 5B and C).

Another limitation concerns the fact that the activity of the Slt2 pathway, which responds to opposing osmotic stress treatments and may modulate intracellular glycerol levels, is ignored in the present study (see Talemi et al. 2016). While the HOG and Slt2 pathway do not seem to directly communicate they may do so via control of glycerol accumulation (see for instance Ahmadpour et al. 2016, Baltanas et al. 2013); the Slt2 pathway hence may affect the behaviour of HOG and especially the two branches when they operate in isolation.

We agree with the reviewer that the Slt2 pathway and the HOG network may affect each other through intermediaries, although Talemi et al. conclude that “it’s mainly the HOG alone mediating adaptation of cellular osmotic pressure for both hyper- as well as hypo-osmotic stress”. Indeed, we have included a Hog1 independent module in our model that could potentially capture any effects of Slt2 and now cite Ahmadpour et al.

*The study is also based on some assumptions that do not seem to be supported by data reported in the literature. For instance (Introduction, seventh paragraph) it has been reported that HOG feedback coincides with the onset of volume recovery, i.e. as soon as the cells start to recover volume, the HOG signal falls rapidly back to basal. This implies, that the sensors sense changes in volume rather than absolute volume. See for instance Babazadeh et al. 2013.*

We believe we have now removed the paragraph to which the reviewer is referring where our choice of wording was poor. As shown both in our data, and in Babazadeh et al., 2013, and by others, altering the dynamics of volume recovery clearly affects Hog1 dynamics.

Results, first paragraph: there are additional single cell studies, some are cited elsewhere, some not: Schaber et al. 2012, Babazadeh et al. 2013, Sharifian et al. 2015 to name a few.

We unfortunately were unaware of some of these papers and now cite them appropriately.

It appears that the authors do not explicitly mention that they chose sorbitol as osmostress agent. Most previous studies employed NaCl. The authors should justify their chose for the agent and the concentrations employed.

Apologies: we have now have added an explanation of our choice of osmotic stress (in the second paragraph of Results). We chose sorbitol because the only known stress sorbitol provides is osmotic. Salts also provide additional stress because cations can be toxic (Posas et al., J Biol Chem 2000; Cyert & Philpott, Genetics 2013). We consider a range of concentrations that give a minimal response to saturated levels of nuclear Hog1 (Figure 2—figure supplement 1; Figure 4—figure supplement 1).

Reviewer #3:

The paper by Granados et al. fundamentally asks the question of how the two branches of the osmo-stress pathways coordinate to produce a rapid, yet accurate, response. The paper presents intriguing data that argues for temporal "hand-over control" from the fast (Sln1/Ssk1) branch to the slow (sho1) branch. As mentioned, the Sln1/Ssk1 is fast and dominates the early response through a bigger share of a common resource of the pathway (PBS2) and when this fast response weans away, PBS2 is liberated for the second branch, the slow one which is now handed the reins. Evidently, the presence of integral control in the slow branch is instrumental to this story, given that it is responsible for matching the perceived stress to the actual stress. As a result, this slow branch might be the major driver of response "accuracy", a hypothesis supported by some data in this manuscript.

I will jump to the bottom line here: Above is the story that I extracted from the paper after reading it multiple times, trying to circumvent distractions to this narrative such as inter-spaced descriptions of step and ramp responses, and also a meager description of the computational modeling, which in my opinion should play a more prominent role in explaining the data. The data is not intuitively obvious, and the paper as written leaves the conclusion to the imagination of the reader. The narrative is ridden with jargon ("accuracy" is such a term), and crucial primary data is not shown (the volume time trajectories, rather a correlation plot between data features that the reader never sees is shown in a major figure that is actually the most important one of this work). There is obviously important information in these data not explicitly shown, since the correlation between volume recovery and Hog1 response seems to be changing with time and the Hog1 response shown for the ssk1 mutant has a rather intriguing biphasic recovery shape at high salt concentration (Figure 3 and accompanying supplementary figures). In light of these and other confusing elements, I found myself entranced by the narrative and model proposed by the authors, but confused as to 1) whether I am interpreting it correcting, or projecting my own partial understanding), 2) if I am understanding it correctly, then the data presented and explanations provided only partially corroborate this model and 3) I truly didn't know what to do with the ramp input data, was it there to make a point that supports the model or to argue that different branches of the pathway are instrumental in different conditions?. So, my rather unorthodox recommendations are:

1) Rewrite the paper to focus on a clear narrative. What is exactly the model that the authors are proposing? Present that clearly (including why the computational model explains the data), and present the data in the order and logical sequence that actually support this model.

We apologize for these difficulties and have almost entirely rewritten the manuscript to focus on a more cohesive narrative and to bring together similar experiments. Additionally, we have included the volume traces, simplified some analyses, and developed a more intuitive, control-theory based model that enables the presentation of clear take-home messages.

2) Present all the data for volume recovery, the same as presenting the data for the Hog1 nuclear residence, not summary statistics of it.

We have now included the time-series data of the volume recovery in Figure 2, Figure 2—figure supplement 1, and Figure 3—figure supplement 1.

3) Bring the computational modeling to the forefront. For example, if the model I extracted out of the paper is correct, then I don't intuitively understand why the Ste11 mutant would have an identical Hog1 response to WT. I would have expected it to have the same initial response, maybe the same or similar Hog1 peak, but then the adaptation phase to be different. Why is it not? I am sure there is an explanation, but it is nowhere to be found in the manuscript. This is not a superficial point, this goes to 1) assessing the rigor and robustness of conclusions and 2) actually giving the reader information that they understand, and therefore trust.

We now explicitly address the different behaviour of the fast mutant and the wild-type both with a new analysis shown in Figure 2 and in our discussion of the new model, with a particular emphasis on the role played by derivative action in the fast pathway. In the response to steps, this difference between the fast mutant and the wild-type is not apparent in the mean-response, but does become clear when single-cells are considered (Figure 2C and D).

Suggestions above only give a partial list of changes that are needed. I intended to write those as a motivation for the authors to take a deeper look at their very intriguing and beautifully collected data, and extract the most robust story out of it.

A few other concrete questions:

1) In Figure 2A, the fast activation Hog1 localization is virtually indistinguishable from the WT; however, in Figure 3A, the fast mutant loses accuracy as the recovery continues. Since the fast mutant's nuclear import of Hog1 is virtually identical to the WT, and synthesis of Gpd1 and Gpp1 has been demonstrated to be the single most important factor towards recovery (Babazadeh et al., 2014), one would expect the resulting glycerol synthesis to be similar, but this argues differently. At what point in the stress perception does this "knee-jerk" reaction fail the cell?

In the new Figure 2D, we show that this loss of accuracy happens at the level of single-cells and is obscured at the population level. We can explain the loss by determining the mutual information between the adaptation time of Hog1 and the level of the stress (Figure 2E). The mutual information becomes lowest for the fast mutant implying that the typical single-cell responses of Hog1 more poorly match the level of stress than the single-cell responses of both the wild-type and the slow mutant.

We can understand when the ‘knee-jerk’ response fails the cell to coincide with the decrease in the magnitude of the derivation action component of the fast pathway. We agree that this is an important point and now discuss it in the second paragraph of the section entitled “The architecture of the HOG network enables both speed and accuracy”.

The authors use the recovery in volume as a proxy for actual stress recovery, but this is the secondary effect from Hog1's interaction with its binding partners and glyercol production. In addition to the change in volume, I would like to see a representative promoter's activity (e.g. pSTL1-YFP) to directly observe where the breakdown in cellular perception occurs. Given the argument that the slow mutant gains in accuracy over time, I would expect to see different promoter dynamics in the two mutants in their optimal environments. This could substantiate the author's claim of the two roles the pathways play.

We agree that the transcriptional dynamics would be interesting and is likely to be environment-specific, but because Hog1 controls multiple mechanisms to respond to osmotic stress we believe that the variable of interest is intracellular glycerol. Given the challenges of measuring intracellular glycerol over time in single cells, cellular volume is, at the moment and in our opinion, the best proxy. Further, the survival data of Figure 6A demonstrates the downstream importance of the differences in dynamics of Hog1 for the two mutants.

2) In Figure 5 (and Figure 5—figure supplement 2), the authors demonstrate an osmostress terrain that should favor the pathway that is able to integrate better – and subsequently demonstrate the "fast" pathway's disadvantage. How were these terrains chosen? Both ramps loosely resemble (and therefore could be interpreted as) ramps, and it's surprising to find the "fast" mutant at nearly a 2X disadvantage (Figure 6A). This may be something more appropriate for the supplemental, but it would be nice to see a more examples of "complex" environments that are more distinct from a classical incline ramp to really cement the accuracy vs. speed argument. What are the corresponding volume (and promoter?) dynamics, and how to they correlate with the Hog1 localization shown in Figure 5D?

Our rational is that a fluctuating ramp should be more deleterious for the fast mutant because its derivative action will respond to each fluctuation in the ramp whereas the slow mutant should not, being both more sensitive to the internal state of the cell and to the history of the input because of its tight coupling to the network’s integral feedback. We have added this explanation to end of the section entitled “Each input pathway favours survival in distinct dynamic environments”. A further advantage of have a fluctuating ramp is that is less likely to activate a confounding hypo-osmotic response in down fluctuations compared to, for example, a fluctuating square wave.

We now included a new analysis of the behaviour in the fluctuating ramps in Figure 4E and F and additional data for fluctuating ramps in the supplement.

3) Mitchell et al. (2015) touched upon how various waveforms are interpreted by the Hog1 pathway (i.e. high frequency is a single step; moderate frequency is a staircase; and low frequency is moderate staircase). This seems very relevant to the model the authors propose; How does the model proposed in Mitchell et al. and their work reconcile?

We believe that our results and model are completely consistent with the findings of Mitchell et al. The different frequencies of the low pass filters present in both the slow and the fast pathway in our model generate behaviour of Hog1 that is consistent with the phenomena found by Mitchell et al. and also by Hersen et al., Proc Nat Acad Sci USA 2008. Further, the correlation between hyperactivation of the network and fitness observed by Mitchell et al. agrees with our interpretation of the survival data, where we expect that excessive activation of Hog1 in the fast mutant leads to a substantial and long-lived overshoot of glycerol, which has a fitness cost in ramps.

4) In the Discussion, the authors state "…this 'passing on' of control occurs predominately through competition for Pbs2." This is a crucial aspect of the model proposed. While this was explicitly modeled, and the resulting Hog1 dynamics matched the predictions, the sharing model of Pbs2 was never experimentally tested. The authors propose that the different spatial localizations of Sho1 and Sln1 could aid in sequestering Pbs2. Since Sho1 localizes to the region of polarized growth, they should tag Pbs2 and Sho1 and observe co-localization at the onset of osmostress. If not possible within a reasonable timeframe, then the authors should spend some time providing evidence from literature supporting this aspect, or if none exists, state that explicitly in order to set the record straight.

We have developed a new control-theory based model which we believe alleviates the anchoring of our results on the competition for Pbs2 because the model relies only on cross-inhibition between the two input pathways and is agnostic to the mechanism.
